# The impact of tiered soft drink taxes in Europe on mean sales-weighted sugar content of soft drinks: a quasi-experimental study

**DOI:** 10.1186/s12889-025-23331-w

**Published:** 2025-06-05

**Authors:** Anna Leibinger, Oliver Huizinga, Karl Emmert-Fees, Sara Pedron, Michael Laxy, Eva Rehfuess, Jacob Burns, Peter von Philipsborn

**Affiliations:** 1 Chair of Public Health and Health Services Research, Institute of Medical Information Processing, Biometry and Epidemiology (IBE), Faculty of Medicine, LMU Munich, Munich, Germany; 2 Pettenkofer School of Public Health, Munich, Germany; 3 German Non-Communicable Disease Alliance (DANK), Berlin, Germany; 4 Professorship of Public Health and Prevention, TUM School of Medicine and Health, Technical University of Munich, Munich, Germany

**Keywords:** Soft drinks tax, Sugar-sweetened beverages, Obesity, Synthetic control

## Abstract

**Background:**

High sugar intake from soft drinks is associated with increased risk of non-communicable diseases. Tiered soft drink taxes applying higher tax rates on beverages with higher sugar content have been used to incentivize producers to reduce sugar content of soft drinks. This study assesses the impact of tiered soft drink taxes in four European countries on the sugar content of soft drinks.

**Methods:**

We used annual sales data from 12 countries from Euromonitor International for 2009 to 2022 to estimate the effect of tiered soft drink taxes in France, Ireland, Portugal, and the United Kingdom (UK) on soft drinks’ mean annual sales-weighted sugar content. We conducted a quasi-experimental study, applying a synthetic control approach in which we used a weighted combination of eight European countries without a soft drink tax serving as control for the four intervention countries.

**Results:**

France, Portugal, and the UK exhibited negative estimated treatment effects, indicating a reduction in average sugar content in these countries. The UK demonstrated the largest estimated effect (-1.7 g sugar/100 ml; 95%-CI: -2.6; -0.8), followed by France (-0.6; 95%-CI: -1.7; 0.4) and Portugal (-0.3; 95%-CI: -1.5; 1.0). Ireland (0.4; 95%-CI: -0.8; 1.7) displayed effects in the opposite direction. Results of the sensitivity analyses indicate that results are robust concerning assumptions underlying the study design and analysis strategy.

**Conclusions:**

Varying effect sizes emphasize the importance of considering specific tax design, co-interventions and contextual factors when implementing tax policies. Further research could help to shed light on these variations and to achieve a higher level of accuracy and precision in the effect estimates.

**Supplementary Information:**

The online version contains supplementary material available at 10.1186/s12889-025-23331-w.

## Introduction

Regular consumption of sugar-sweetened beverages (SSB) increases the risk for overweight, obesity, and diabetes mellitus [1–3]. SSB consumption has increased markedly worldwide over the past decades and is considered a key driver of the global epidemic of obesity and diabetes mellitus [4, 5]. Various policies to reduce SSB consumption and its adverse health effects exist, including limits on the sale of SSB in schools, nutrition labeling, and marketing restrictions [6]. Among these, taxes on SSB have gained particular prominence, and as of October 2024, such taxes have been implemented in more than 90 countries worldwide [7] (for a definition of different tax designs and other key definitions, see Panel 1).

Many expert groups, including the World Health Organization (WHO), recommend the implementation of SSB taxes on health grounds [8–10]. Specifically, in its guideline on fiscal policies to promote healthy diets published in June 2024, WHO strongly recommends the implementation of policies to tax SSB, citing evidence that such taxes lead to reductions in SSB purchases, likely to translate into reduced consumption and finally improved health [10]. Additionally, SSB taxes may have additional benefits. The WHO guideline notes that tiered taxes (i.e., taxes levied at higher rates on products containing more sugar), in particular, can incentivize manufacturers to reduce the sugar content of SSB and consumers to choose products with less sugar. It found, however, that current evidence from policy evaluations was insufficient to issue recommendations on specific policy design elements, such as a tiered tax design [10].

The current evidence base on the specific effects of tiered SSB taxes is indeed limited, but growing. The systematic review on which the WHO guideline is based included six studies (five from the UK and one from Portugal) that reported the impact of tiered soft drink taxes on sugar content [11]. All six studies observed evidence of reformulation, but only one study conducted statistical tests, and none weighted the sugar content of SSB by sales volumes [11]. In fact, weighting sugar content by sales, i.e. weighting products sold in larger volumes more heavily than less consumed products, allows for a more accurate reflection of average population-level exposure than measures of sugar content not weighted by sales [12]. Further studies examined the effect of the tiered SSB tax introduced in the UK in 2018 on the volume of beverages with varying sugar content, finding that sales and purchases shifted towards beverages with lower sugar content [13, 14]. A more detailed description of these and further studies on tiered SSB taxes is provided in Sect. 3. of the supplementary material. To the authors’ knowledge, no comparable studies have been conducted on tiered SSB taxes introduced in other countries.

To address this evidence gap, the present study uses a quasi-experimental study design to evaluate the effect of tiered soft drink taxes in four countries (France, Ireland, Portugal, and the UK) on the sales-weighted sugar content of soft drinks sold in these countries.

**Table:** 

***Panel 1: Key definitions***
**Tax types** • **Excise taxes** are levied on specific products, typically at the point of manufacture or distribution. They can either be based on volume (beverage volume or sugar content) or on the product value (ad valorem) [15]. • **Ad valorem taxes** are calculated based on the price of the product [15]. • **Volumetric taxes** are calculated based on the volume of the product [15].
**Tax structures** • **Flat taxes** apply the same tax to all soft drinks regardless of their sugar content [15]. • **Tiered taxes** apply different tax rates depending on the sugar content of soft drinks. Typically, they apply higher tax rates to beverages with higher sugar content [15].
**Products considered in this study** • **Soft drinks** are defined in the present study as all non-alcoholic and non-dairy beverages that are sweetened with sugar or other caloric or non-nutritive (artificial) sweeteners. This definition includes sugar-sweetened beverages, beverages sweetened exclusively with non-nutritive sweeteners, and beverages that use a combination of the two. • **Added sugars** are defined by the European Food Safety Authority as sucrose, fructose, glucose, starch hydrolysates (glucose syrup, high-fructose syrup) and other isolated sugar preparations added during food and drink preparation and manufacturing. The term “sugars” refers to any monosaccharides and disaccharides [16].

## Methods

### Study design overview

This study examined the impact of tiered soft drink excise taxes on the mean sales-weighted sugar content in soft drinks using a synthetic control (SC) approach– a quasi-experimental method that enables the evaluation of population-level health interventions with aggregated, observational data. Quasi-experimental studies are commonly used as an alternative for establishing causal impacts when randomized controlled trials are not feasible [17]. To do so, these approaches estimate a counterfactual, which represents a hypothetical alternative scenario in which no intervention (i.e., in our case, no tiered excise tax) is implemented. We applied the SC approach to four countries individually, all of which implemented a tiered soft drink tax in 2017 or 2018: France, Ireland, Portugal, and the UK. As controls, we employed other European countries that do not have any soft drink tax in place. We used data from the Euromonitor Passport database to calculate the yearly mean sales-weighted sugar content as our outcome.

### Intervention and control countries

Eight European countries had implemented tiered excise taxes on soft drinks on a national level as of December 2023, when we conducted this analysis (Croatia, France, Hungary, Ireland, Latvia, Poland, Portugal, and the UK). Of these, we excluded four countries as they introduced tiered soft drink taxes after 2020 and therefore lacked sufficient post-intervention data. Specifically, we excluded Croatia and Poland which introduced their tiered tax in 2020 and 2021, respectively [18, 19], and Latvia and Hungary, which switched from a flat tax to a tiered tax system in 2022 [20, 21]. The Spanish region of Catalonia implemented a tiered tax on soft drinks in 2017 [22], however, we did not include it due to a lack of data at the sub-national level. The remaining four countries that have a tiered tax on soft drinks– France, Ireland, Portugal, and the UK– served as our intervention countries.

As control countries, we selected eight European countries (Austria, Denmark, Germany, Greece, Italy, Netherlands, Sweden, and Switzerland) that currently do not have a soft drink tax in place and that are geographically, economically, and culturally similar to the intervention countries. A detailed description of this selection process of the control countries can be found in Sect. 4.b. of the supplementary material.

### Description of the intervention

#### Intervention design

**Fig. 1:**
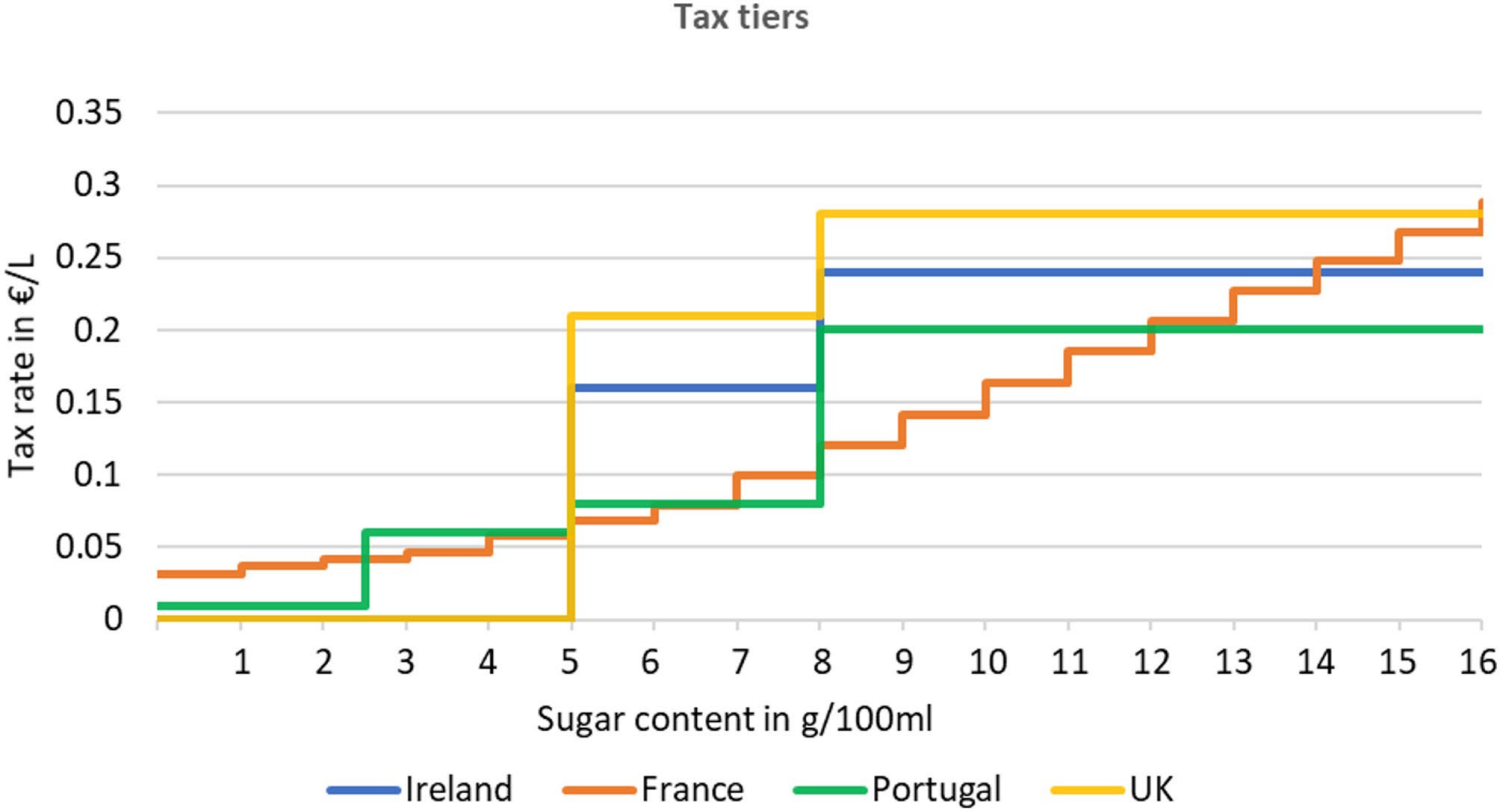
Tax rates of tiered soft drink taxes in France, Ireland, Portugal and UK. Notes: References: [23–26]. The tax rates in the UK are 18p and 24p/L, here displayed in Euros with an exchange rate of 1.16 EUR/GBP as of May 30, 2023

A graphical representation of the soft drink tax tiers in each of the four intervention countries is provided in Fig. 1. Both the UK and Ireland have two tiers; however, the UK has higher tax rates than Ireland [23, 24]. Portugal implemented four tiers with a relatively low tax rate [25]. France applies a higher tax rate for each additional gram of sugar, employing a more continuous design and lower tax rates compared to the two tiers in the UK [26]. A detailed description of the design and timing of the intervention in each country is provided in Sect. 2. of the supplementary material.

### Intervention timing

For any quasi-experimental study evaluating changes over time, it is important to determine the timing of the intervention, i.e., the point at which an effect due to the intervention is expected. Usually, policy decisions on soft drink taxes are announced 1–2 years before the implementation of the tax [15]. Even before that, policymakers may circulate the notion, and lobbyists and industry experts may know that a tax is under consideration. In anticipation of the tax, some companies may already start reformulating their products at this stage [15, 27]. To capture this anticipatory effect, for countries that announced the tax during the first half of the year we designated the year prior to the announcement as the first post-intervention year. If the announcement was made in the second half of the year, we set the year of the announcement as the first post-intervention year. Consequently, for France, 2017 was the first post-intervention year; for Ireland and Portugal, it was 2016; and for the UK, 2015 was the first post-intervention year.

### Data sources and methods of assessment

This study used data by Euromonitor International, a market research company. Euromonitor provides sales and ingredients data from primary and secondary data sources such as company reports, official statistics, store audits, product information (such as ingredient and nutrient declarations), interviews with company representatives, and estimates by industry experts [28]. For soft drink sales, Euromonitor covers both off-trade sales (i.e., sales through retail outlets) and on-trade sales (i.e., through hospitality and catering outlets). The Euromonitor database includes yearly aggregated country-level industry data on usage of ingredients, such as various kinds of added sugars, in different categories of beverages, as well as volume sales data for the same beverage categories. Combining the ingredients and sales datasets allows for the calculation of a mean sales-weighted sugar content of soft drinks per country. We used annual data from 2009 to 2022, as 2009 was the first year for which comparable and harmonized data on the sugar content of soft drinks were available.

### Outcome

Our outcome was the mean sales-weighted sugar content of soft drinks. The following soft drinks categories were included in our analysis: carbonates, flavored and functional bottled water, energy and sports drinks, juice drinks (up to 24% juice), nectars, and ready-to-drink tea. The following ingredients were included as sugars: sucrose, fructose, dextrose, glucose/fructose syrup, glucose/corn syrup, high fructose corn syrup, and invert sugar. For glucose/fructose syrup, glucose/corn syrup, high fructose corn syrup, we assumed a 70% sugar content, as explained in Sect. 4.e. of the supplementary material. Further details are provided in Sect. 4.f.-g. of the supplementary material. We chose the mean sales-weighted sugar content of all soft drinks (including those artificially sweetened) and not only of sugar-sweetened beverages to be able to capture not only industry reformulation but also a shift in consumption towards beverages containing non-sugar sweeteners in order to estimate population-level exposure. Further, the introduction of new products or the discontinuation of existing products can also influence the mean sales-weighted sugar content, depending on the market share of the respective products.

### Statistical methods

The SC represents a data-based approach to constructing a comparable control from a donor pool, i.e., from multiple potential controls. From this donor pool, the SC approach creates a counterfactual for the time after the intervention, i.e., it estimates how outcomes would have developed had the intervention not been implemented. It does so by creating a weighted average of the control units from the donor pool that mimics the pre-intervention outcome trend of the intervention unit as closely as possible and subsequently projecting that trend into the post-intervention period [17, 29]. A key strength of the SC approach is its emphasis on achieving a close pre-intervention fit between the intervention unit and the synthetic control, which enhances the credibility of causal inference when evaluating interventions where randomization is not possible. Given that underlying trends may differ across regions, the SC method is especially appropriate for this study, as it minimizes bias by ensuring that comparisons are made between units with highly similar pre-treatment trends, i.e., in this study, between intervention countries and their respective synthetic controls. In this study, we used an R package that applies the generalized synthetic control method using a regression-based approach. This approach is designed to minimize the influence of unobserved factors that vary over time and across countries. As described above, the design of the tax varies among the four intervention countries with regard to the timing, the number of tiers, and the tax rate. We therefore applied the SC approach to each intervention country separately. We used all eight control countries that were selected for the study (selection process described above) in the donor pool. Based on a linear interactive fixed effects model, we compared the counterfactual created from this donor pool to the actual outcome in the respective intervention country. This allowed for estimation of a treatment effect for each post-intervention year (i.e. the difference between the estimated counterfactual outcome and the observed outcome in the intervention country in that year) as well as average treatment effect across all post-intervention years (i.e. the average of all of the year-specific treatment effects post-intervention).

In order to assess the short-term effect, the long-term effect, as well as the average effect of each tax, we focused on the effect after year 1, the effect after the final post-intervention year, and the average treatment effect, respectively. To increase the robustness of our analysis, i.e. to ensure that the specific choice of study design did not lead to spurious findings, we conducted a secondary analysis using a controlled interrupted time series (cITS) approach. Details on the methods and results of the secondary analysis are presented in Sect. 4.d. and 5.b. of the supplementary material.

#### Sensitivity analyses

We conducted five different kinds of sensitivity analyses. First, we delayed the timing of the expected intervention effect by one year, to test that no effect is detected at a time when no effect should be expected. Second, we verified that no single country in the control group was overly influential to the SC model by running the SC analysis several times, each time leaving out one country from the control group. Third, we randomly selected three countries from the control group and applied our model to each of these with 2016 as the hypothetical first post-intervention year, as if they were an intervention country, to verify that no intervention effect could be seen in these three countries.

Fourth, we conducted the SC analysis assuming a sugar content of glucose/fructose syrup, glucose/corn syrup, and high fructose corn syrup of 80% instead of 70%. Fifth, we conducted the SC analysis with a traditional SC approach as a post-hoc analysis. There are multiple statistical software options for implementing the synthetic control approach, each of which uses different techniques for estimating the weights that are used to calculate the synthetic control from the control countries. As described above, the generalized SC approach we applied uses a linear interactive fixed effects technique to calculate the weighted average of the control unit outcomes. The traditional SC approach relies on arithmetic weighted averages and thus represents a simpler alternative to the generalized SC approach. To assess whether our choices in implementing the SC approach had an influence on the findings, we also employed another R package, which applies a traditional SC approach, as a post-hoc sensitivity analysis.

We conducted all data processing and analyses in R version 4.2.1. For the SC approach, we used the Generalized Synthetic Control Method (“gsynth”) package [30] in the main analysis and the Augmented Synthetic Control Method (“augsynth”) package [31] for the post-hoc sensitivity analysis. For the cITS approach, we applied the Linear and Nonlinear Mixed Effects Models (“nlme”) package [32].

## Results

### Descriptive trends in sales-weighted sugar content across European countries

**Fig. 2:**
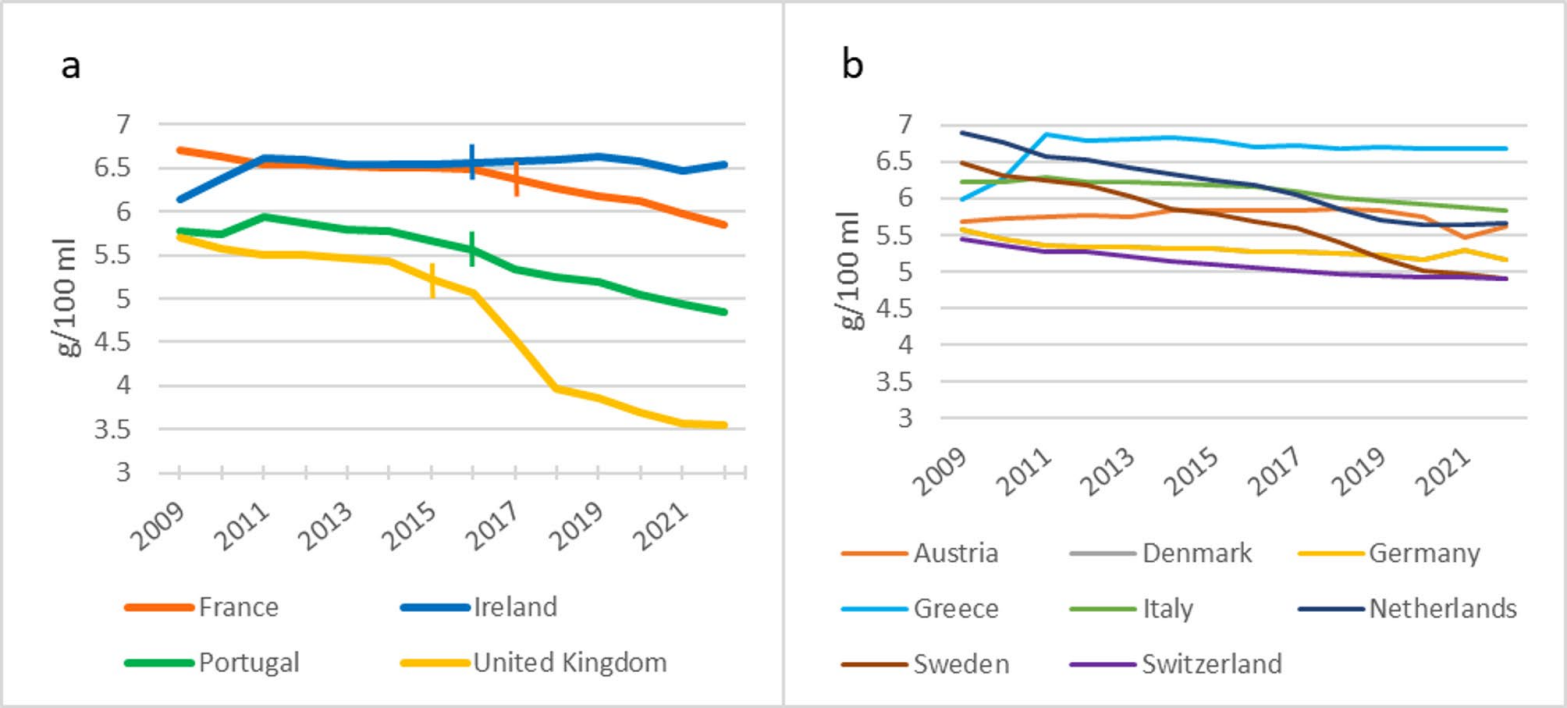
Sales-weighted sugar content in g/100 ml, 2009–2022. Notes: Panel (**a**) shows the four intervention countries and (**b**) the eight control countries. The vertical marks in panel a) denote the time of intervention as defined in this study

The sales-weighted sugar content decreased in all four intervention countries following the tax announcement. The UK exhibited the most substantial difference, with a 34.6% reduction from the last pre-intervention year (2014) to 2022. Similarly, Portugal showed a sizable drop of 14.4% between 2015 and 2022, while France experienced a decrease of 9.9% from 2016 to 2022. Conversely, Ireland demonstrated a comparatively minimal change, with a decrease of 0.1% from 2015 to 2022. All eight control countries also showed a decline in sales-weighted sugar content between 2015 and 2022– on average, a reduction of 6.9%, ranging from 1.4% in Greece to 15.5% in Sweden (see Fig. 2).

### Main analysis results: SC approach

**Fig. 3:**
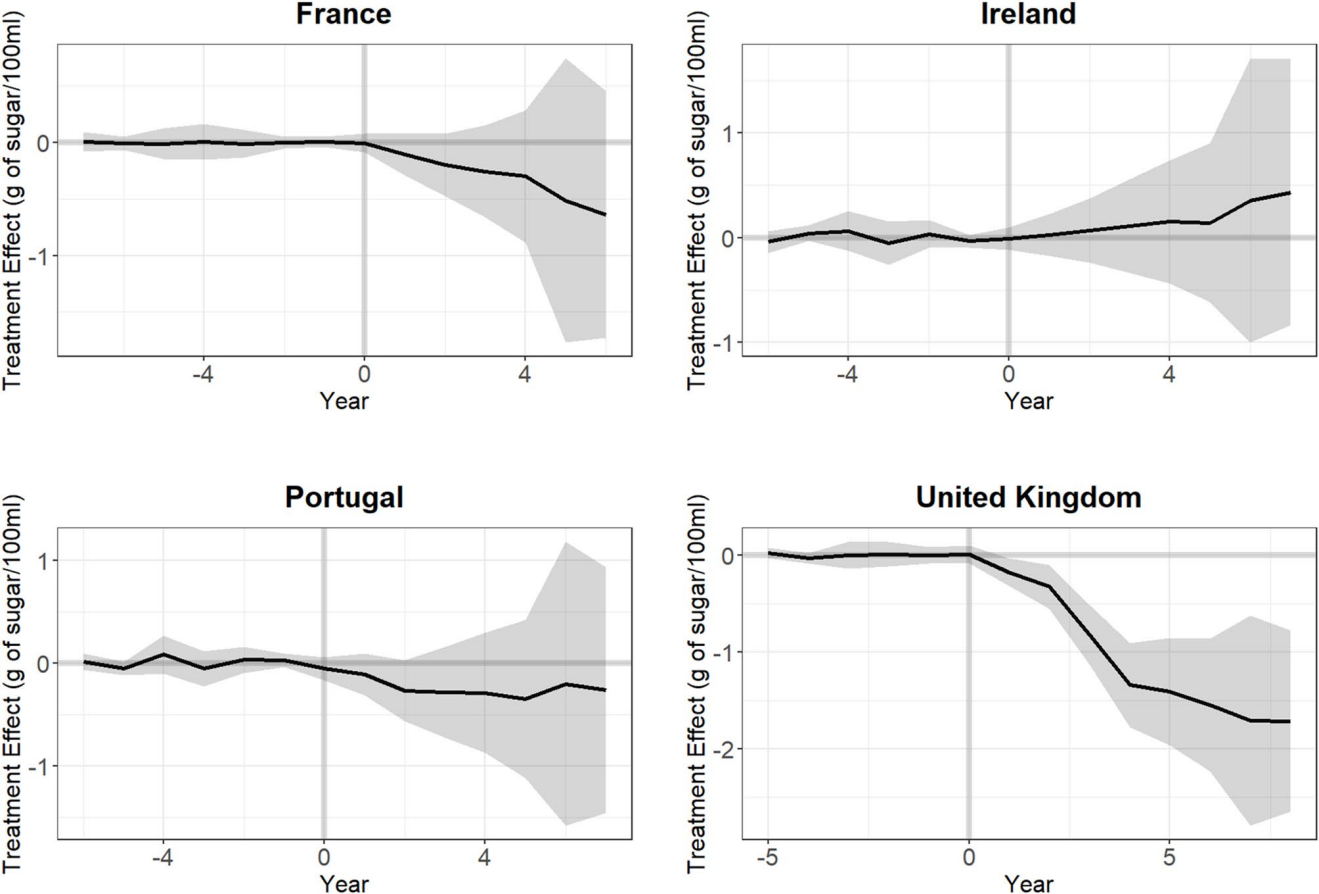
Results of the synthetic control analysis. Notes: Treatment effects pre- and post-intervention. Year 0 denotes the last pre-intervention year. The grey area denotes the 95% confidence interval

The estimated treatment effects are shown in Table 1; Fig. 3. For France, Portugal, and the UK, the estimated treatment effects for year one and the last year, as well as the estimated average treatment effects were negative, indicating a reduction in observed sugar content compared to the counterfactual synthetic control. The most substantial treatment effect was observed in the UK, with an estimated treatment effect of -1.71 g of sugar/100 ml (95%CI: -2.61; -0.82) in year eight after the intervention. In France, the treatment effect in year six after the intervention was − 0.64 g of sugar/100 ml (95%CI: -1.67; 0.40), which was comparatively smaller than in the UK. Meanwhile, Portugal exhibited the smallest treatment effect of the three at -0.26 g of sugar/100 ml (95%CI: -1.51; 0.98) in year seven after the intervention. All three of these countries displayed smaller treatment effects in year one than in the last year. In contrast, Ireland showed a treatment effect in the opposite direction, as the treatment effect in year 7 after the intervention was 0.43 g of sugar/100 ml (95%CI: -0.84; 1.71), meaning that, relative to the control countries, sugar content in Ireland increased post-intervention. As indicated in Fig. 3; Table 1, and evident from the reported confidence intervals, the UK is the only country where statistical significance at the 95% confidence level was reached.

**Table 1:** Results of synthetic control analysis: estimated treatment effects of tiered soft drink tax on mean sales-weighted sugar content

Year after intervention	Treatment Effect*	95%-CI	p-value
**France**			
1 year	-0.10	[-0.28; 0.07]	0.24
6 years	-0.64	[-1.67; 0.40]	0.23
Average Treatment Effect	-0.33	[-0.89; 0.22]	0.24
**Ireland**			
1 year	0.03	[-0.19; 0.24]	0.82
7 years	0.43	[-0.84; 1.71]	0.50
Average Treatment Effect	0.18	[-0.48; 0.84]	0.59
**Portugal**			
1 year	-0.11	[-0.32; 0.10]	0.30
7 years	-0.26	[-1.51; 0.98]	0.68
Average Treatment Effect	-0.25	[-0.90; 0.40]	0.45
**UK**			
1 year	**-0.17**	[-0.31; -0.03]	0.02
8 years	**-1.71**	[-2.61; -0.82]	< 0.001
Average Treatment Effect	**-1.13**	[-1.60; -0.66]	< 0.001

Bold: denotes statistical significance at an alpha level of 5%. Control Countries: Austria, Germany, Netherlands, Denmark, Greece, Italy, Sweden, Switzerland. *in g of sugar/100 ml

To assess the quality of the pre-intervention fit, we visually inspected the alignment between the intervention unit and the synthetic control. A graphical representation of the pre-intervention fit is provided in eFigure 2 of the supplementary material.

Results of the secondary analysis revealed a negative trend change in all four intervention countries. In line with the main analysis, this effect was largest for the UK, followed by Portugal, France and Ireland. Statistical significance was reached for the former three.

### Sensitivity analyses results

Delaying the intervention time by one year resulted in slight variations in the magnitude and significance levels of the treatment effects and trend changes, providing no clear indication for determining the optimal intervention time. Leaving out one country at a time from the control group showed similar trends as in the main analysis with some variation in the magnitudes of the treatment effects and their confidence intervals. In most cases, the estimated average treatment effect was relatively stable. Further, the three randomly selected control countries we tested the method on were Denmark, Germany, and Italy. As hypothesized, no significant reformulation trend was discernible in any of these three countries. The only statistically significant effect we observed was a minor positive trend change in Germany in the cITS approach. Results of this sensitivity analysis using the SC approach are summarized in Table 2 and a more elaborate version that also includes the cITS approach is available in eTable 10 of the supplementary material.

**Table 2:** Sensitivity analysis, applying the SC approach to randomly selected control countries

	ATE*	95%-CI	p-value
**Germany**			
	-0.04	[-0.60; 0.53]	0.90
**Denmark**			
	-0.10	[-0.70; 0.50]	0.75
**Italy**			
	-2.04E-01	[-0.77; 0.36]	0.48

Abbreviations: CS: Synthetic control; ATE: Average treatment effect (across all post-intervention periods). *in g of sugar/100 ml

Finally, applying a sugar content of 80% to glucose/fructose syrup, glucose/corn syrup, and high fructose corn syrup in the SC approach, showed very similar results to the main analysis, as summarized in eTable 12 of the supplementary material.

Results of the post-hoc sensitivity analysis using the “augsynth” R package showed negative treatment effects in the UK and Portugal, whereas Ireland and France showed positive treatment effects. Similar to the main analysis, these results were only statistically significant for the UK. Further details on the results of the sensitivity analyses are reported in Sect. 5.c. of the supplementary material.

## Discussion

### Summary of key findings

This study examined the impact of tiered soft drink taxes on the mean sales-weighted sugar content of soft drinks in four countries (France, Ireland, Portugal, and the UK). We used a synthetic control approach to estimate the treatment effect of the tax on sugar content relative to a counterfactual scenario based on data from eight European countries without a soft drink tax. For France, Portugal, and the UK we found negative estimated treatment effects, indicating that the tiered soft drink taxes in these countries had the desired effect of reducing the average sugar content of soft drinks sold in these countries. The largest effect was observed in the UK, with an estimated treatment effect of -1.71 g of sugar/100 ml in year eight after the intervention. Portugal and France showed smaller effects, which did not reach statistical significance at the 5% level. Ireland did not exhibit any meaningful reduction in mean sugar content compared to the control countries. The findings from the secondary analysis, the cITS, were mostly in line with those of the main analysis, with no findings that would suggest alternative conclusions.

### Comparison of results with other studies

Several studies have examined the impact of tiered soft drink taxes on sugar content, each contributing evidence through different methodological approaches. For instance, Public Health England reported a 28.8% reduction in sales-weighted sugar content between 2015 and 2019 in the UK, based on a before-and-after comparison of consumer data [12]. For the same time period, Scarborough et al., using an interrupted time series design, estimated a 33.8% point drop in the proportion of drinks exceeding 5 g of sugar per 100 ml in the UK [14]. Bandy et al. found a 34% reduction of sales-weighted sugar content in the UK from 2015 to 2018 using a before-and-after approach on the basis of product-level nutrient data and sales volumes to calculate sales-weighted sugar content [33]. Allais et al. provided complementary evidence from two countries that introduced a tiered tax, showing a 31% reduction in the sugar content of newly launched beverages in the UK, and a more modest 6% reduction in France in a difference-in-difference analysis [34]. Further details and additional studies from Portugal, Poland, and South Africa are summarized in the supplementary material. While each study uses different data sources and study designs, and some analyze only a certain subgroup of soft drinks, they all point to reductions in sugar content following the introduction of a tiered tax, contributing to a growing body of evidence. Taken together, these studies support and contextualize our findings, with each approach having distinct strengths and limitations.

### Interpretation of results and policy implications

In line with previous studies [12–14, 33, 35], our results suggest that tiered soft drinks taxes can be effective in reducing the sugar content of soft drinks, but that effects may vary substantially. Of the various factors that may moderate the effect of tiered soft drink taxes on sugar content of soft drinks, our subsequent discussion centers on intervention design, co-interventions, and contextual factors likely to play a key role in shaping industry and consumer response and therefore observed effects. Ongoing policy debates illustrate the importance of these considerations. For instance, in the United Kingdom, the government is currently reviewing its tax design and is considering potential adjustments [36].

#### Tax design

The specific design of the tax may have contributed to the stronger estimated effects in the UK. Research suggests that the higher the tax rate, the more likely that producers will reformulate [34]. Further, the larger the steps between different tax tiers, the stronger the incentive for the industry to reformulate in order to reach the next lower tier [34]. The granular tax tiers of the French tax design may therefore be less effective than designs that exhibit larger steps, such as the one implemented by the UK [37, 38]. Additionally, the comparatively low tax rates may have further limited the impact of the French tax [38, 39]. Contrastingly, the higher tax rates in the UK in comparison to the other three countries may have motivated the industry’s willingness to reformulate its products. While the UK, France, and Ireland have not changed the tiered tax design since implementation, Portugal has continuously adjusted tax rate and tiers over the last few years [40] which may have limited the size of the effect.

#### Co-interventions

Effects on the sugar content of soft drinks in the UK may also have been strengthened by co-interventions. Alongside the soft drinks tax, the UK government implemented non-binding agreements with food industry actors aspiring to a reduction in the sugar content of foods by 20% between 2015 and 2020. Additionally, the introduction of the UK soft drink tax was accompanied by an extensive public awareness campaign, along with heightened media focus on the adverse effects of sugar, all possibly contributing to the decreasing sugar content of soft drinks after 2015 [33]. To the authors’ knowledge, no comparable co-interventions were implemented in France, Ireland and Portugal.

#### Context and policy process

A number of factors related to context and the policy process may explain why in France and Ireland, estimated effects were smaller than in the UK. Of the four intervention countries in our study, France stands out as it is the only country that already had a flat soft drink tax in place before implementing a tiered tax [41]. It is also the only one of the four that taxes artificially sweetened beverages, (albeit at a comparatively lower rate [26], which may have weakened incentives for producers to reduce sugar content by replacing sugar with low-calorie sweeteners. Additionally, France exhibits much lower consumption levels of soft drinks than most other European countries [42, 43], which may have resulted in a demand less sensitive to price changes [39]. This may have made it preferable for producers to pass the tax through to the consumers instead of reformulating the product [42, 43], and may have limited shifts to lower-sugar beverages taxed at lower rates.

Ireland already had downward trends in sugar-sweetened beverage sales many years before announcing the soft drink tax [44]. Several sources, including the Irish State Department of Health, indicate that the tax may have resulted in substantial reformulation very early in the policy process, starting years before the official announcement of the tax [45–48]. This may have limited the scope for further reformulation after the implementation of the tax and may have limited our ability to detect any such reformulation.

#### Market structure

Depending on factors that define the role of a producer in a given market, the profit-generating potential of specific commercial strategies may vary. Responses of individual producers to soft drink taxes may therefore vary considerably between countries depending on such factors as their product portfolio, their brand strength, their position in the market or the behavior of competitors. These dynamics may impact the outcome in each country considerably [49].

### Strengths and limitations of this study

Our study has a number of strengths. To the best of our knowledge, no other studies to date have assessed the impact of tiered soft drink taxes on sales-weighted sugar content of soft drinks in Europe using a controlled study design (an overview of existing research is given in Sect. 3. of the supplementary material). We employed two approaches—SC and cITS—which yielded consistent results. Our analyses are based on a pre-registered protocol, and all differences between the protocol and our final study are explained in Sect. 6. of the supplementary material [50].

Our study also has limitations. While our design is generally well-suited for policy evaluation, it cannot fully eliminate the effects of confounding variables. Policies introduced concurrently, for example, may have biased our results. The proprietary nature of the data and our reliance on estimates for certain sugar content calculations introduced uncertainty. Moreover, the uncertain timing of interventions and the use of aggregated annual data limited the precision of our analysis. We also did not differentiate between different soft drinks categories or brands. While this was not a direct limitation of our study’s primary objectives, it may limit the understanding of the mechanisms and heterogeneity behind the observed effects.

Another limitation is the potential confounding impact of the COVID-19 pandemic, which may have affected countries differently due to variations in lockdown measures and consumer behavior. However, our outcome data do not suggest a major disruption during the observation period coinciding with the COVID-19 pandemic, as we do not observe any substantial shocks or abrupt changes in sugar content or soft drink sales that would indicate strong COVID-19-related effects. Nevertheless, we acknowledge that unobserved pandemic-related factors could have influenced the results to some extent.

A further limitation of our analysis is that we are unable to examine heterogeneity in treatment effects across the different tax tiers. Specifically, while taxation effects may differ depending on the sugar content of individual products, our data do not provide information on the distribution of products across different tax tiers. As a result, we cannot assess whether the effect of sugar taxes varies by sugar content level. Nevertheless, the mean sales-weighted sugar content remains a meaningful and policy-relevant outcome measure, as it reflects the overall public health impact of sugar reduction efforts across the soft drink market.

Our outcome variable of sales-weighted sugar content allowed us to capture overall population-level exposure to added sugar through soft drinks. However, this approach meant that we could only estimate the combined effect of industry reformulation and some shifts in consumption patterns. Reducing sugar intake from soft drinks can occur through four primary pathways: (1) reformulation of existing soft drinks to contain less sugar, (2) introduction of new, lower-sugar products and/or discontinuation of high-sugar products, (3) shifts in consumption patterns away from high-sugar products (possibly increasing intake of artificial sweeteners), and (4) an overall reduction in the total volume of soft drinks consumed. Our outcome measure—mean sales-weighted sugar content—captures the first three pathways, as they affect the sugar content of soft drinks and/or the relative sales share of high-sugar to low-sugar soft drinks sold. However, if consumers reduce their overall soft drink consumption uniformly across products (i.e., without a relative shift towards lower-sugar options), such changes would not be reflected in our measure. Thus, while our outcome is well-suited to assess reformulation and relative shifts, it does not capture reductions in soft drink consumption per se. Indeed, research has shown that in Ireland sugar intake from soft drinks decreased after the introduction of its soft drinks tax (even though our analysis did not show a statistically significant effect on the sugar content of soft drinks) [51]. Due to these limitations, results of our analysis should be interpreted with caution.

### Implications for research

The variation in effect sizes observed between the four countries examined in our study suggests that intervention design, co-interventions, and contextual factors may influence the effects of tiered soft drink taxes on sugar reduction. The role of these factors should be explored in further research. Such research should ideally be based on less aggregated outcome data to improve the precision of results. Among others, such research should use more detailed data to investigate heterogeneity in taxation effects across different sugar content tiers and/or relating to the proximity of products to tier thresholds. Analyzing how tax responses vary by tier and by proximity to thresholds would provide valuable insights into the effectiveness of tiered soft drink taxes and could inform the strategic design of tax thresholds, when governments try to optimize the tax design, as is currently happening in the UK [36]. Such analyses could help policymakers set tiers that maximize incentives for reformulation and sugar reduction, thereby increasing the public health impact of soft drink taxation. Further, future research could assess the validity of studies evaluating the impact of soft drink taxes by analyzing trends in other sugary product categories beyond soft drinks. This would help assess whether the observed effects are specific to soft drink taxation or reflect broader, secular shifts toward reduced sugar consumption. Additionally, there is a need for rigorous evaluations of the long-term impact of tiered soft drink taxes on a broader set of outcomes, including industry practices such as marketing efforts, the amount and the use of the tax revenue generated, equity effects, and overall dietary patterns, as noted elsewhere [52]. Additionally, the use of non-sugar sweeteners for reformulation has been a growing concern and should be further researched, as policies that are solely focused on reducing sugar content may lead to higher consumption of non-nutritive sweeteners with potential adverse effects on health [53, 54]. All this could contribute to the evidence base for designing and implementing effective policies to address the public health challenges associated with excessive sugar consumption through soft drinks.

## Conclusions

Tiered soft drink taxes can be effective in reducing the average sugar content of soft drinks subject to the tax, but effects may vary depending on multiple factors, including intervention design, co-interventions, and contextual factors. In light of this evidence, as well as the well-established effectiveness of soft drink taxes in reducing soft drink consumption and associated harms, tiered soft drink taxes should be considered by policy makers as part of comprehensive strategies to improve population nutrition.

## Electronic supplementary material

Below is the link to the electronic supplementary material.

Supplementary Material 1

## Data Availability

The data used in this study is proprietary and owned by Euromonitor International, a market research company. Access to the data can be acquired from Euromonitor International.
