# Supplementary material for: The impact of tiered soft drink taxes in Europe on mean sales-weighted sugar content of soft drinks: a quasi-experimental study
Source: BMC Public Health. 2025 Jun 5;25:2106. doi: 10.1186/s12889-025-23331-w (PMC12142870; doi:10.1186/s12889-025-23331-w)
Supplement: Supplementary file 1 — Supplementary Material 1 [file 12889_2025_23331_MOESM1_ESM.docx]

Supplementary material

Supplementary material to: Leibinger, A., Huizinga, O., Emmert-Fees, K., Pedron, S., Laxy, M., Rehfuess, E., Burns, J., von Philipsborn, P. *The impact of tiered soft drink taxes in Europe on mean sales-weighted sugar content of soft drinks: a quasi-experimental study.* Manuscript submitted to BMC Public Health, 2024.

**Table of content**

[1. Overview of soft drink taxes in Europe 3](#_Toc197113708)

[2. Description of interventions and policy processes in intervention countries 5](#_Toc197113709)

[France 5](#_Toc197113710)

[Ireland 5](#_Toc197113711)

[Portugal 6](#_Toc197113712)

[UK 6](#_Toc197113713)

[3. Overview of and comparison with similar studies focusing on reformulation 6](#_Toc197113714)

[a. United Kingdom 7](#_Toc197113715)

[b. Portugal 8](#_Toc197113716)

[c. Poland 8](#_Toc197113717)

[d. South Africa 8](#_Toc197113718)

[e. Studies that involve several countries 8](#_Toc197113719)

[4. Detailed description of methodology and data sources 9](#_Toc197113720)

[a. Information searches on the interventions under consideration 9](#_Toc197113721)

[b. Description of selection process of control countries 9](#_Toc197113722)

[c. Synthetic control weights 10](#_Toc197113723)

[d. Detailed description of the statistical method of the controlled interrupted times series (cITS) analysis 10](#_Toc197113724)

[e. Data sources 11](#_Toc197113725)

[f. Category definitions 11](#_Toc197113726)

[i. Beverage categories 12](#_Toc197113727)

[ii. Ingredients categories 16](#_Toc197113728)

[g. Calculation of variables 17](#_Toc197113729)

[h. R-code of the statistical analyses 17](#_Toc197113730)

[5. Additional results 21](#_Toc197113731)

[a. Full results and further graphics of SC analysis 21](#_Toc197113732)

[b. Results and graphics of cITS 24](#_Toc197113733)

[c. Sensitivity analyses 26](#_Toc197113734)

[6. Differences between protocol and manuscript 38](#_Toc197113735)

[7. Reporting guideline checklist 39](#_Toc197113736)

[References 45](#_Toc197113737)

# Overview of soft drink taxes in Europe

As of December 2023, 11 countries in Europe have implemented a soft drink tax. Eight adopted a tiered tax and the remaining three a flat tax. Additionally, the region of Catalonia in Spain also has a tiered soft drink tax in place. The following eFigure 1 and eTable 1 give a more detailed overview of the different tax designs.


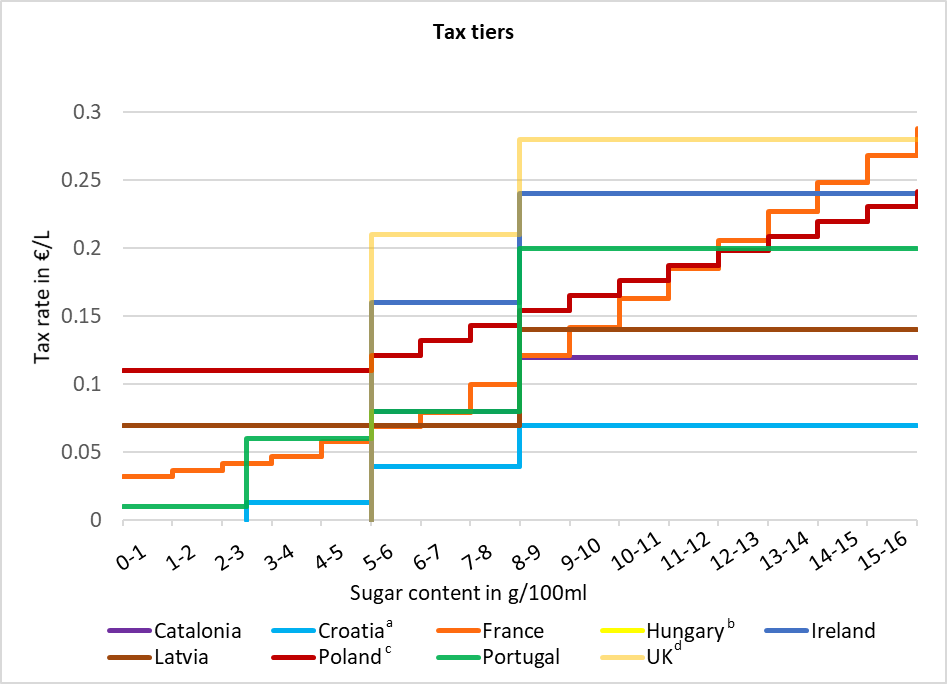


***eFigure 1:*** ***Tiered soft drink tax rates of European Countries.****^a^The tax rates in Croatia are 0.1, 0.3 and 0.6 Kuna/L, here displayed in Euros with an exchange rate of 0.13 EUR/HRK as of July 13, 2023. ^b^Hungary is not displayed in the graph, as the tax does not only depend on sugar content, but also on the product type as indicated by the tariff number. The rates in Hungary span from 0.021 EUR/L to 0.062 EUR/L. ^c^The tax rate in Poland is 0.5 PLN below 5 g of sugar/100 ml and an additional 0.05 PLN with each gram of sugar, here displayed in Euros with an exchange rate of 0.23 EUR/PLN as of July 13, 2023. ^d^The tax rates in the UK are 18p and 24p/L, here displayed in Euros with an exchange rate of 1.16 EUR/GBP as of May 30, 2023. Data sources: [1–8]*

| **eTable 1: European countries with soft drink taxes as of December 2023** | | | | |
| --- | --- | --- | --- | --- |
| **Country** | **Tax type** | **Tax design** | **Date of introduction** | **Date of revision** |
| **Belgium** | Flat tax | €0.068/L excise on soft drinks with added sweeteners; €0.41/L and €0.68/100 kg excise on liquid and powder concentrates, respectively | January 2016 | n.a. |
| **Croatia** | Tiered tax | HRK 0/L on drinks with ≤2 g/100 ml;  HRK 0.1/L (€0.013/L) on drinks 2–5 g/100 ml;  HRK 0.3/L (€0.04/L) on drinks 5–8 g/100 ml;  HRK 0.6/L (€0.07/L) on drinks >8 g/100 ml;  Syrups and concentrates are similarly taxed at four rates based on sugar content. Higher tax rates are levied on energy drinks containing methylxanthine or taurine. | April 2020 | n.a. |
| **Finland** | Flat tax | €0.22/L on sugar-containing soft drinks and €0.12/L on sugar-free soft drinks and mineral waters | 1940 | 2011 |
| **France** | Tiered tax | Since 2018:  €0.03/L for sugar content <= 1 g per 100 ml  sliding tiers up to  €0.24 for sugar content > 14 g/100 ml and <= 15 g/100 ml  €0.02 for each additional gram above 15 g/100 ml  From January 2012 to 2018:  flat tax of €0.11 per 1.5 L ($0.08 per L) on drinks with added sugars or artificial sweeteners. | January 2012 | July 2018 |
| **Hungary** | Tiered tax | Since July 2022:  HUF 8-23/L (€0.021-€0.061) on soft drinks (depending on sugar content and tariff number)  HUF 105-310/L (€0.28-$€0.83) on syrup concentrates (depending on tariff number);  From 2011 to 2022:  HUF 7/L (€0.02) on soft drinks; HUF 200 /L ($0.62) on syrup concentrates. | 2011 | July 2022 |
| **Ireland** | Tiered tax | € 0.16/L for sugar content >= 5 g/100 ml and < 8 g / 100 ml  € 0.24/L for sugar content >= 8 g/100 ml | May 2018 | n.a. |
| **Latvia** | Tiered Tax | Since 2022:  €0.07/L for sugar content= < 8 g/100 ml  €0.14/L for sugar content > 8 g/100 ml  For non-alcoholic beverages containing other sweeteners, the excise duty rate will remain the same as for other non-alcoholic beverages until now, namely, € 0.07/L of beverage.  From 2004 to 2022:  €0.074/L excise on drinks with added sugar, sweetener, or other flavoring (excludes fruit/vegetable juices with <10% added sugar and flavored/functional waters without added sugars, sweeteners, or flavorings).  Implemented May 2004; increased tax rate 2016. | May 2004 | 2022 |
| **Poland** | Tiered tax | PLN 0.5/L (€0.11) on soft drinks with added sweeteners, caffeine, or taurine  PLN 0.05 (€0.01) extra fee per gram of sugar >5 g/100 mL  PLN 0.09 per L (€0.02) for drinks containing caffeine or taurine. Total soda fee cannot exceed PLN 1.2 ( 0.27). Excludes sports or juice drinks with <5g sugar/100mL and dairy drinks. Drinks with >20% juice content and >5g sugar/100mL are not charged the base fee of PLN 0.5 (€0.11) per L. | January, 2021 | n.a. |
| **Portugal** | Tiered tax | €0,01/L for sugar content < 2.5 g/100 ml  €0.06/L for sugar content >= 2.5 g/100 ml and <= 4.9 g/100 ml  €0.08/L for sugar content >= 5 g/100 ml and <= 7.9 g/100 ml  €0.20/L for sugar content >= 8 g/100 ml Applies to non-alcoholic beverages; exempts milks (cow or substitute) and 100% juices | February 2017 | 2022 |
| **Spain** | Flat tax | National tax: 21% VAT (increase from 10%) on drinks containing added natural and derived sweeteners and/or sweetening additives, excluding dairy. Regional tax in Catalonia: €0.08 per L for 5–8 g sugar/100 mL, €0.12 per L for drinks with added sugars and >8 g sugar/100 mL, implemented in May 2017. | January 2021 | n.a. |
| **United Kingdom** | Tiered tax | £0.18 (€0.21)/L for sugar content > 5 g/100 ml and <= 8 g/100 ml  £0.24 (€0.28)/L for sugar content > 8 g/100 ml  Exempts dairy drinks (>75% dairy) and 100% fruit/vegetable juices. | April 2018 | n.a. |
| *Countries in the EEA + UK and Switzerland without SSB taxes: Austria, Bulgaria, Cyprus, Czech Republic, Denmark, Estonia, Germany, Greece, Iceland, Italy, Liechtenstein, Lithuania, Luxembourg, Malta, Netherlands, Norway (previously existing tax abolished with effect on January 1, 2021), Romania, Slovakia, Slovenia, Sweden, and Switzerland. Exchange rates as of July 14, 2023. Data sources: [1–8]* | | | | |

# Description of interventions and policy processes in intervention countries

### France

France initially introduced a relatively low flat tax on beverages that are sweetened with sugar or artificial sweeteners in 2011 [9]. In 2017, a new government was elected and announced in September of that year that they would revise this flat tax into a tiered tax with the aim to tackle business practices instead of consumer behavior. The new, tiered tax came into effect in July 2018 [9] and was last revised in January 2021 [4]. The new tax starts at 3.2 cents per liter for soft drinks containing between 0 and 1 g of sugar per 100 ml and goes up with each additional gram of sugar. Beverages that contain between 14 and 15 g of sugar are taxed with 24 cents per liter. For each gram above 15 g of sugar, an additional 0.02 cents per liter is charged. France also taxes all soft drinks containing artificial sweeteners with an additional 3.17 cent per liter [4]. France thus stands out as a unique case due to its pre-existing flat tax model, its use of granular tax tiers, and its taxing of artificially sweetened beverages.

### Ireland

The publication of the EU Action Plan on Childhood Obesity and the introduction of soft drink taxes in some European countries spurred public debate around a soft drink tax in Ireland in 2015 [10, 11]. As a result, the finance department announced in September 2016 that such a tax would be included in the Finance Act of 2017. The plan was finalized in October 2017 with the official announcement of the 2018 budget that included such a tax. The tax, consisting of two tiers, was then implemented in May 2018 [10, 11]. Ireland taxes soft drinks that contain between 5 and 8 g of sugar per 100 ml at 16 cents per liter and drinks above 8 g of sugar per 100 ml at 24 cents per liter [7].

### Portugal

In Portugal, the first soft drink tax was announced in December 2016 and went into effect in February 2017 [12]. Initially, this tax consisted of two tiers with a cut-off at 8 g of sugar/100 ml (€ 0.08/L in the lower tier and € 0.16 in the higher) [12]. However, effects of this tax on reformulation and sugar consumption were not at the level that the Portuguese government was hoping for, and the tax was therefore expanded into four tiers with the tax in the highest tier increasing iteratively over the course of five years (from 2018 to 2022) [5, 12, 13]. The latest adjustment happened in June 2022 and set the cutoffs for the tiers at 2.5 g, 5 g, and 8 g of sugar per 100 ml. The tax rates range from 1 cent per liter for beverages with sugar content below 2.5 g per 100 ml, over 6 and 8 cents per liter in the middle tiers, to 20 cents for beverages containing more than 8 g of sugar per 100 ml [5]. The continuous changes make it particularly challenging to specify the intervention timing.

### UK

The British government first announced plans for a soft drink tax in March 2016. One year later, the final draft of the legislation was introduced, and the two-tiered tax was implemented in April 2018 [14]. The UK taxes soft drinks with sugar content between 5 and 8 g of sugar per 100 ml at 18 pence (21 Euro cents) per liter and beverages with more than 8 g of sugar per 100 ml at 24 pence (28 Euro cents) per liter [6].

# Overview of and comparison with similar studies focusing on reformulation

There have been numerous studies that measure the impact of soft drink taxes on consumption and sales [15–19], pricing [17, 20–23], or health outcomes [24–27]. However, fewer studies have analyzed the impact of tiered soft drink taxes on sugar content, and the ones that did, often used data that were not sales-weighted or they applied only a pre-post-comparison without controlling for any underlying trends. In the following, we will discuss studies that analyzed the impact of a tiered soft drink tax on sugar content and compare them with our study.

## United Kingdom

An evaluation of the tiered soft drink tax in the UK by Public Health England found that there was a 28.8 % reduction in sales-weighted average sugar content per 100 ml between 2015 and 2019 [28]. Data for this analysis originated from Kantar Worldpanel’s take-home consumer panel and different consumer surveys. This study does not account for underlying trends or counterfactual scenarios, but simply measures the difference in sugar content pre- and post-intervention [28]. This may explain why the sugar reduction shown in this study is larger in magnitude than the treatment effect estimated in our analysis.

Similarly, Bandy et al. found that the sales-weighted sugar content of soft drinks decreased by 34 % from 4.4 g/100 ml in 2015 to 2.9 g/100 ml in 2018 in the UK. They also employed a cross-sectional pre-post-analysis, using nutrient composition data collected online, paired with volume sales data offered by companies [29]. Hashem et al. employed a comparable study design with a cross-sectional pre-post-analysis, collecting the data of soft drink products manually in nine large supermarket chains in the UK in 2014 and 2018, which did not allow for the calculation of a sales-weighted outcome variable [30]. The average sugar content of the 83 products that they obtained data for in both years decreased by 42 % from 9.1 g/100 ml to 5.3 g/100ml [30].

Scarborough et al. employed an interrupted time series approach to measure the impact of the tiered soft drink tax in the UK on sugar content. Their outcome was the proportion of available drinks with sugar content greater than 5 g/100 ml, which had fallen by 33.8 percentage points from 2015 to 2019. They collected data from the websites of leading supermarkets in the UK at 85 time points. Due to the different outcome measure, their findings are not directly comparable to the ones in our study; they are, however, in line with a general trend of substantially decreasing sugar content of soft drinks in the UK, as observed in our study [31].

Chu et al. analyzed the sugar content of fruit juice, juice drinks, and smoothies specifically targeted at children before and after the introduction of the tiered soft drink tax in the UK. They included 131 fruit juices, juice drinks and smoothies in their study, of which only seven were eligible for the tax. Of these seven, four were reformulated by September 2018, resulting in a sugar content below the tax threshold of 5 g of sugar per 100 ml [32]. This study’s aim was to show that the tax should be redesigned in order to target more of these beverages that are targeted at children, which is why their sample of tax-eligible beverages was too small to draw any general conclusions about reformulation [32].

Even though none of the aforementioned studies are directly comparable in both outcome and methodology to our study, they all display the same trend and underscore our findings that the tiered soft drink tax has had a substantial impact on sugar content of soft drinks in the UK.

## Portugal

When evaluating Portugal’s tiered soft drink tax, Goiana-da-Silva et al. did not measure reformulation directly, but triangulated this outcome by evaluating the reduction of volume of beverages consumed in the highest tax tier as well as the reduction of sugar consumption from soft drinks eligible for the tax [33]. As both of these decreased significantly, by 41 % and 15 % respectively, they assumed that this must be partially due to reformulation, which is in line with our findings [33].

## Poland

Wierzejska assessed the impact of the tiered soft drink tax that was implemented in Poland in January of 2021 [3]. They collected nutrition label data of soft drinks in five large supermarkets in Warsaw before and after the implementation of the tax. The average sugar content of the beverages in their sample decreased by 19.8 % from 8.6 g/100 ml to 6.9 g/100 ml. However, the average sugar content in this study was not sales-weighted and the study design only allowed for a non-parametric test of the difference in pre- and post-intervention sugar content [3]. As more data become available, further research will be needed to measure the impact of the Polish tax more rigorously.

## South Africa

South Africa implemented a tiered soft drink tax in April of 2018 that taxes beverages with a sugar content above 4 g/100 ml and increases with each additional gram of sugar. An initial assessment of this tax by Stacey et al. in 2019 revealed evidence of product reformulation in response to the tax. Many brands adjusted their products to contain less than 4 g of sugar per 100 ml [34]. The study identified significant reductions in sugar content, with numerous products that initially had over 10 g of sugar per 100 ml being reformulated to contain less than 5 g [34]. These findings indicate that a granular tax design increasing with every gram of sugar, which is similar to the one in France, can have a meaningful impact on the sugar content of soft drinks.

## Studies that involve several countries

Allais et al. conducted a controlled difference-in-difference study assessing the impact of the tiered soft drink taxes in the UK and France by assessing the sugar content of new sugar-sweetened beverages that were launched between 2010 and 2019. They observed a significant reduction of sugar content in new beverages of 31% between 2015 and 2019 after the implementation of the tiered tax in the UK. In France, they found a significant reduction of 6% between 2017 and 2018 after the implementation of the tiered tax; however, there was no significant reduction in 2019 compared to 2017 in France. While their outcome was average sugar content of new beverages and not of the whole market, their findings are in line with ours, as they found a larger and more sustained impact in the UK than in France [35].

# Detailed description of methodology and data sources

## Information searches on the interventions under consideration

To ascertain the timing and the characteristics of the interventions, we conducted a search of scientific and grey literature on the timing of the announcement and implementation of soft drink taxes and their tax design in our intervention countries. To identify relevant sources, we used Google and Google Scholar. We also searched government websites for information on the implementation of soft drink taxes and the tax design. Our search included the use of keywords including “soft drink tax”, “sugar-sweetened beverage tax”, “tiered”, “tax design”, “announcement”, “implementation”, “timing”, “policy”, and the country names. The results of these searches, including a description of the policy process in each intervention country, are summarized in Chapter 2 of this supplementary material.

## Description of selection process of control countries

In order to identify control countries that are geographically, economically and culturally similar to the intervention countries, we considered countries in the north, west, and south of Europe. This included five EU countries in Southern Europe (Greece, Italy, Malta, Cyprus, and Spain), five EU countries in Western Europe (Austria, Belgium, Germany, Luxembourg, and the Netherlands) [36], five Scandinavian countries (Denmark, Finland, Iceland, Norway, and Sweden), and Switzerland.

From these 16 countries, we excluded Cyprus, Iceland, Luxembourg, and Malta, as for these countries only modeled data are available from Euromonitor (i.e., calculated data based on data collected in other countries). Of the remaining 12 countries, three (Belgium, Finland, and Spain) have a flat tax and were excluded [37]. Norway had previously implemented a flat tax that was discontinued in 2021. It was therefore excluded, as the tax was still in place for most of this study’s timeframe [38]. The remaining eight countries (Austria, Denmark, Germany, Greece, Italy, Netherlands, Sweden, and Switzerland) that have never had and currently do not have a soft drink tax in place served as controls.

## Synthetic control weights

eTable 2 displays the weights that were assigned to each control country in order to mimic the intervention country as closely as possible pre-intervention. These weights were then applied to the post-intervention data points to create the counterfactual that was subsequently compared to the observed outcome in the intervention country.

| ***eTable 2:*** ***Weights assigned to each control country for each of the four synthetic control analyses*** | | | | |
| --- | --- | --- | --- | --- |
| **Control countries** | **Intervention countries** | | | |
|  | **France** | **Ireland** | **Portugal** | **UK** |
| **Austria** | -1.545 | 0.517 | 0.575 | -0.841 |
| **Denmark** | -2.112 | 0.900 | 1.191 | -0.636 |
| **Germany** | 1.041 | -0.218 | -0.113 | 0.914 |
| **Greece** | -3.591 | 1.013 | 0.940 | -2.456 |
| **Italy** | -0.347 | 0.279 | 0.470 | 0.243 |
| **Netherlands** | 2.098 | -0.154 | 0.370 | 2.598 |
| **Sweden** | 1.343 | 0.213 | 0.891 | 2.491 |
| **Switzerland** | 1.117 | -0.149 | 0.057 | 1.206 |

## Detailed description of the statistical method of the controlled interrupted times series (cITS) analysis

The difference between the cITS and SC approaches relates to how the control countries are modeled. While the SC approach constructs a weighted average of control countries, the cITS approach treats each control country equally. In our cITS analysis, we used a linear model fitted with the generalized least squares method. Results of the cITS provide estimates for the level change, i.e., the immediate change in the outcome after the intervention in the intervention country relative to the control countries, and the trend change, i.e., the change in the slope post-intervention relative to the trend change of the control countries [39, 40]. A detailed description of the cITS approach is provided in the supplementary material. The regression equation of the cITS analysis had the following form:

*sugar_jkt_ = β_0_ + β_1_ year + β_2_ country_k_ + β_3_ year_t_*country_k_ + β_4_ post_jt_ + β_5_ year_post_jt_ + β_6_ country_k_*post_jt_ + β_7_ country_k_* year_post_jt_ + ε_t_*

where *sugar* is the outcome (i.e., sales-weighted sugar content) at time t, in country k, and at intervention status j; *year* is the time in years elapsed since the beginning of the study period (i.e., since 2009); *year_post* is the time in years elapsed since the intervention; *country* is a dummy variable that takes the value of 0 for the control countries and 1 for the intervention country; *post* is a dummy variable, taking on the value of 0 pre-intervention and 1 post-intervention. *β_6_ and β_7_* represent the two effect estimates of interest for the tax: *β_6_* represents the level change, i.e., the immediate change in the outcome after the intervention in the intervention country relative to the control countries, and *β_7_* the trend change, i.e., the change in the slope post-intervention relative to the trend change of the control countries [39, 40].

## Data sources

We used data from the “market size” component of the Euromonitor Passport database for our study. Specifically, we used total volume market size in order to calculate sales volume, including both off-trade sales (i.e. sales through retail outlets, such as supermarkets, non-grocery retailers, vending machines, internet retailing, and home delivery services) and on-trade sales (i.e. through hospitality and catering outlets, such as restaurants, bars, cafés, and hotels) [41]. In order to calculate sugar sales from soft drinks, we used data from the “ingredients” component of the Euromonitor Passport database. As described above we used total volume data, including on- and off-trade sales. Euromonitor collects this data through sales data, ingredient declarations on the packaging, and, when these are not available, on patent literature, trade interviews, and the knowledge of its technical specialists [41]. For three of the eight control countries (Greece, Sweden, and Switzerland) data for 2022 were projected by Euromonitor. To calculate the mean sales-weighted sugar content of soft drinks, we combined data from the market size and the ingredient volume database. A list of sources used by Euromonitor is available from their website [41].

We assumed a conservative average sugar content of 70% for glucose/corn syrup, glucose/fructose syrup, and high fructose corn syrup. The German Directive on Sugars for Human Consumption requires that glucose syrup have a minimum dry mass of 70% [42], whereas the dry mass of glucose/fructose syrup is not regulated, though products on the German market typically range between 75–85% [43].This conservative estimate might lead to an underestimation of the actual sugar content and sugar sales from soft drinks. The reason for this is that the volumes of glucose/corn syrup, high fructose corn syrup, and glucose/fructose syrup mentioned in ingredient declarations may pertain only to the sugar content of these syrups, excluding the water content.

## Category definitions

In the following, category definitions of the soft drinks categories and the sugar types used in our analysis are given.

### Beverage categories

Category definitions were provided by Euromonitor International.

| **eTable 3: Included and excluded beverage categories and their definitions** | | | |
| --- | --- | --- | --- |
| **Hierarchy**  **level** | **Passport Category** | **Included?** | **Category definition** |
| 0 | Softdrinks | No | This is the aggregation of the following categories; Carbonates, Fruit/vegetable juice, Bottled water, Functional drinks, Concentrates, RTD tea, RTD coffee and Asian speciality drinks. |
| 1 | Bottled Water | No | This category is the aggregation of still bottled water (spring, mineral & purified), carbonated bottled water (spring, mineral & purified), flavored bottled water and functional bottled water. Bulk bottled water is split out separately. It is defined as packaged drinking water – purified, spring or mineral – that is packaged in a container of 8 litres or larger. The bulk bottled water data types apply to Still Bottled Water and Carbonated Bottled Water but do not apply to functional or flavored bottled water. Bulk water is classified according to the new Bulk retail volume and Bulk retail value data type regardless of channel of sale: bulk water sold via retail locations and bulk water delivered direct to a consumer’s home is included. Bulk water sold to institutions (offices, schools etc.) remains excluded. |
| 2 | Flavored Bottled Water | Yes | Combined total of, both sparkling and still flavored bottled water. However, functional flavored or functional carbonated water is tracked in functional water. Commonly fruit juice or essence has a content of one milligram per liter or less. Fruit essence is sometimes referred to as ‘natural flavors’. Products with significant percentage juice content (often listed on the packaging) should be included in Juice Drinks (up to 24% juice). In addition, flavored bottled water does not normally contain colorings or significant amounts of added sweeteners. So-called ‘infusions’ – still water containing small pieces of fruit for added flavor – would be tracked here. |
| 2 | Functional Bottled Water | Yes | This subsector utilizes production techniques which go beyond typical water purification processes. Functional bottled water will often include added vitamins, minerals, fruits or herbs. This category includes all functional bottled water, both carbonated and still. The subsector will primarily include nutraceutical or fortified waters, where various types of fruit or herbal concentrate or vitamin and mineral extracts are added to the bottled water for nutrient value. The product typically carries added calcium and vitamins, or added herbs, such as ginseng or gingko biloba. ”Fitness” waters, which typically contain electrolytes and amino acids as well as added vitamins and minerals, are included here. Sport and Energy drinks are excluded. These products often do not contain coloring, and tend to be a lighter drink than sports drinks such as Gatorade, which often feature higher added sugar or electrolyte content. Leading brands in off-trade volume include SmartWater and Propel Fitness Water. Products with both significant amounts of carbohydrate (such as dextrose or glucose) and electrolyte, and which are marketed as recovery beverages should be tracked in Sports Drinks, regardless of product name. |
| 1 | Carbonates | Yes | Sweetened, non-alcoholic drinks containing carbon dioxide are included here. All carbonated products containing fruit juice (“sparkling juices”) are included here, unless they are tea-based (these are included in carbonated RTD tea) or carbonated Energy drinks, which are included in Energy Drinks. Carbonated bottled water is also excluded. Carbonates are an aggregation of cola carbonates and non-cola carbonates, whether regular or low calorie. Euromonitor International includes both naturally and artificially-sweetened carbonates. |
| 2 | Cola Carbonates | Yes* | This is the aggregation of regular cola and low-calorie cola carbonates. Colas generally include some combination of caramel color, caffeine, and a sweetener. While vanilla and the kola nut were originally used to provide the characteristic flavor and caffeine content, other flavoring and caffeinating ingredients are now commonly used. |
| 3 | Low Calorie Cola Carbonates | Yes* | All products that have, and which are marketed on the basis of having, lower calorie content than regular cola carbonates are included here. This is regardless of whether they contain artificial sweeteners, sugar, both or alternatively neither. This is the aggregation of standard low calorie cola carbonates and speciality low calorie cola carbonates. |
| 3 | Regular Cola Carbonates | Yes* | This is the aggregation of standard regular cola carbonates and speciality regular cola carbonates. |
| 2 | Non-Cola Carbonates | Yes* | Refers to carbonated soft drinks which are not cola carbonates, including carbonated fruit juice. However, all carbonated waters, RTD teas, sports drinks, energy drinks, and RTD coffees are excluded. This is the aggregation of lemonade/lime, orange, mixers, and other non-cola carbonates. Brewed soft drinks, such as Fassbrause or maltas, are included here. |
| 3 | Lemonade/Lime | Yes* | This is the aggregation of juice-based and non juice-based lemonade-lime non-cola carbonates. |
| 3 | Ginger Ale | Yes* | Carbonated beverage made with ginger or ginger flavoring. Common varieties are Canada Dry, Schweppes and Seagram’s. |
| 3 | Tonic Water/Other Bitters | Yes* | Tonic water is a carbonated beverage that derives its distinct bitter taste from the addition of quinine. This also includes tonic waters that are lightly flavored (for example tonic water with lemon). Common varieties are Canada Dry, Schweppes and Seagram’s. Tonic water with lemon or lime or orange flavor added is known as bitter lemon or bitter lime or bitter orange, respectively. Also includes non-alcoholic carbonated bitter aperitifs such as Crodino or Sanbitter. Common brands are Canada Dry and Schweppes. |
| 3 | Orange Carbonates | Yes* | This is the aggregation of juice-based and non-juice-based orange non-cola carbonates. |
| 3 | Other Non-Cola Carbonates | Yes* | Includes all carbonated soft drinks that are not included in regular cola carbonates, low-calorie cola carbonates, lemonade-lime, orange or mixers. Products featuring flavor mixes, such as orange-pineapple, are included here. Leading brands in off-trade volume include Mountain Dew, Dr Pepper and Shasta. |
| 1 | Concentrates | No | This is the aggregation of liquid concentrates and powder concentrates. |
| 2 | Liquid Concentrates | No | Concentrates and syrups, or alternatively known squashes or dilutables, which are diluted with water before consumption. Based commonly on fruit juices, however are also available as other in other forms, for example cola. Dilution ratios vary from country to country, due to local preferences and available brands. Leading brands in off-trade volume include Robinsons, Tucan, and Brookes. |
| 2 | Powder Concentrates | No | Powder concentrates, including granules and blocks/bars/cubes are diluted with water before consumption. Please note that powdered ice teas are included within this subsector. Leading brands in off-trade volume include Tang, Nestea Instant Iced Tea Mix, and Nestlé Orange-C. |
| 1 | Juice | No | This category covers all still packaged juice obtained from fruits or vegetables by mechanical processes, reconstituted or fresh, often including pulp or fruit/vegetable puree. All unpackaged juices are excluded. Only still drinks are included here. Carbonated varieties are included non-cola carbonates. Juice-flavored milk drinks and fruit shakes which are primarily milk are excluded–these are instead tracked in Packaged Foods Dairy. However, if the juice component is greater, the product is to be excluded from Packaged Foods Dairy coverage and tracked under the relevant category (based on % juice content) within Soft Drinks juice. This sector is the aggregation of 100% juice, nectars (25-99% juice content), juice drinks (up to 24% juice content), and coconut & other plant waters. |
| 2 | 100% Juice | No | This is the aggregation of not from concentrate 100% juice, reconstituted 100%juice and frozen 100% juice. |
| 3 | Not from Concentrate 100% Juice | No | 100% pure fruit or vegetable juice that has not been reconstituted from concentrate. These products are commonly freshly-squeezed and stored within chilled cabinets. Not from concentrate juices can be partially pasteurized, which means their shelf life can range from four days to three weeks. Leading brands in off-trade volume include Tropicana Pure Premium Orange Juice, Florida’s Natural Orange Juice and Simply Orange. |
| 3 | Reconstituted 100% Juice | No | 100% pure fruit or vegetable juice (still) that has been reconstituted from concentrate. These products are normally ambient/room temperature. They usually have an extended shelf life, commonly up to 12 months. Leading brands in the off-trade include Minute Maid Premium Orange Juice, Dole, and Fruktovy. |
| 2 | Juice Drinks (up to 24% Juice) | Yes | Includes all still juice drinks made up of fresh juice or concentrate, not exceeding 24% juice content. Leading brands in off-trade volume include Minute Maid, Capri Sun, and President. |
| 2 | Nectars | Yes | This category includes all frozen and unfrozen juices that are manufactured using a base of concentrated juice or a pasteurized purée of the fruit pulp, to which sugar and water are/can be added. For citrus fruits, the fruit content of nectars is usually over 50%, however can go as low as 25% for other fruits. Leading brands in off-trade volume include Hui Yuan, Ocean Spray, and Nongfu Orchard. |
| 2 | Coconut and Other Plant Waters | No | This category contains packaged beverages fully or partially derived from the liquid contained in coconuts or root-based plants/vegetables. In addition to coconut water, other examples include birch water, cactus water, artichoke water, maple water and other light, clear liquid beverages derived from water-bearing plants. In general, these products are lighter and can be consumed for hydration or sometimes post-athletic recovery (in contrast to thicker, heavier juice products). Coconut milk and plant-based dairy substitutes are excluded. Some coconut or other root plants are combined with other fruits in blended juice beverages. If coconut water or other plant waters represent the largest ingredient in the juice combination (70% coconut water v 30% orange juice, for example), the product will be tracked here. Otherwise, the product will be tracked in Nectars or Juice Drinks. Unpackaged coconut water and other unpackaged plant waters are excluded. Brand examples: Vita Coco, Innocent coconut water. |
| 1 | Energy Drinks | Yes | These are functional drinks designed to boost energy levels. Often lightly carbonated, these contain high levels of caffeine and a number of added water-soluble vitamins, most often a selection of B vitamins including niacin, pantothenic acid, vitamin B6, and vitamin B12. Ingredients can also include amino acids such as taurine and glucuronolactone, as well as herbal products such as guarana and ginseng. Can be carbonated or still. Leading brands in off-trade volume include Red Bull, Monster and RockStar. Glucose-based energy beverages such as Lucozade Energy are included here. |
| 2 | Reduced Sugar Energy Drinks | Yes* | Includes products with lower sugar content compared to ‘standard’ offering, and is positioned on the basis of being ‘better for you’/reduced sugar. A sugar-reduced product will typically contain artificial sweeteners in addition or instead of sugar, and as a result the total sugar/calorie content is lower compared to what is considered ‘standard’. |
| 2 | Regular Energy Drinks | Yes* | Energy drinks not meeting the definition of “reduced sugar energy drinks” provided above. |
| 1 | Sports Drinks | Yes | The choice of sports drink usually depends on the provision of fluids, carbohydrates or both. Included into this subsector are isotonic, hypotonic and hypertonic sports drinks. Isotonic are products that replace lost body fluids, electrolytes (sodium, potassium and chlorides) and glucose in similar concentrations to existing body fluid without causing either swelling or shrinkage of cells. These products usually contain about 5-8% carbohydrate and are intended to be consumed during exercise and/or heat exposure. Hypotonic this product is a weaker solution than your body fluid. These drinks contain less carbohydrate and therefore have lower osmolality (fewer dissolved particles than blood). These drinks help the body to speed up water absorption and are best used when you need urgent fluid replacement, as in after exercise. These drinks are not the best for energy replacement. Hypertonic - this drink is a stronger solution than your body fluid. These drinks are designed to replace and maintain energy levels during exercise of at least one hour. They are absorbed slowly and therefore are not appropriate for fluid replacement. Leading brands in off-trade volume include Gatorade, Powerade, and Aquarius. |
| 1 | RTD Coffee | No | Includes packaged ready-to-drink coffee, consumed either hot or cold, made using a base of either brewed coffee or coffee extract. Excludes all coffee flavored milk drinks that primarily target children, or where coffee is one of a number of flavors within the brand range. Leading brands in off-trade volume include Georgia, Nescafé, and Suntory Boss. |
| 1 | RTD Tea | Yes | This category includes all packaged products based on brewed tea or tea extract. May be sweetened or unsweetened, carbonated or still, with a wide variety of different flavorings. May contain juice. |
| 2 | Carbonated RTD Tea and Kombucha | Yes* | Carbonated packaged ready-to-drink tea, this does not include leaf or powdered tea. Naturally carbonated RTD Teas, such as kombucha, are included here. Leading brands in off-trade volume include Lipton Ice Tea Sparking, Rosynka, and TEBS. |
| 2 | Still RTD Tea | Yes* | Non-carbonated packaged ready-to-drink tea this does not include leaf or powdered tea. Leading brands in off-trade volume include Master Kong Green Tea, Lipton, and Nestea Lemon Tea. |
| 2 | Asian Speciality Drinks | No | This category includes all traditional Asian drinks not included in RTD tea or juice drinks, including products such as Bandung (rose syrup with milk), bird’s nest, tamarind juice, ginger, lemongrass, roselle, zalaka, jelly drinks including grass jelly (cincau), sugar cane, and vinegar drinks. Lactic acid drinks, such as Calpis, are included here. Drinks containing a limited amount of yogurt (generally 3% or less) such as Bikkle, are included here, though drinking yogurts such as Yakult are excluded. While both products are highly popular in markets like Japan, drinking yogurts will contain mostly yogurt with a very short shelf life (two weeks or less), while yogurt drinks will contain less than 3% dairy and remain on the shelves for up to 9 months. All nut or pulse-based products, such as peanut milk, almond juice, or soy drinks are tracked in Non-Dairy Milk alternatives in Passport Packaged Food. |
| *Included as part of the category of next higher hierarchy level (e.g. “Cola carbonates” are included in our analysis as part of the category “Carbonates”); Data sources: Euromonitor International (Passport database). | | | |

### Ingredients categories

Category definitions were provided by Euromonitor International.

| **eTable 4: Included and excluded sugar-containing ingredient categories and their definitions** | | | |
| --- | --- | --- | --- |
| **Hierarchy level** | **Passport category** | **Included?** | **Category definition** |
| 2 | Sugars and Bulk Sweeteners | No | These are the nutritive or bulk sweeteners, which add bulk and texture to food and drink as well as sweetening capability. The category includes simple sugars (monosaccharides), processed sugars and syrups and sugar alcohols (derived from the hydrogenation of sugar and syrups). |
| 3 | --Dextrose | Yes | Dextrose, an isomer of glucose, is a simple and naturally occurring sugar and one of the main two sugars found in honey. It is produced commercially by the enzymatic hydrolysis of starch. Main product categories include soft drinks, baked goods and snack bars. |
| 3 | --Fructose | Yes | A simple sugar (monosaccharide) found in honey, fruits and vegetables. |
| 3 | --Glucose/Corn Syrup | Yes* | A naturally occurring, simple sugar (monosaccharide), glucose is produced commercially through enzymatic hydrolysis of starch. As corn syrup, it is most widely used as a thickener or humectant and is also used in conjunction with high intensity sweeteners. Key product categories include confectionary, dairy products and ice cream. |
| 3 | --Glucose/Fructose Syrup | Yes* | A naturally occurring, simple sugar (monosaccharide), glucose is produced commercially through enzymatic hydrolysis of starch. As fructose syrup, it is most widely used as a replacement for sugar in dairy products, ice cream and biscuits. |
| 3 | --High Fructose Corn Syrup | Yes* | This is derived from corn syrup that has been enzymatically treated by the enzyme glucose isomerase to increase the fructose content before being mixed with pure corn syrup (100% glucose). This process increases sweetness and HFCS has the same sweetness as sugar. It is mainly used in soft drinks, but also dairy products and jams and preserves. |
| 3 | --Invert Sugar | Yes | Invert sugar, usually used as syrups, are sucrose-based syrups treated with the glycoside hydrolase enzyme invertase, and/or an acid, which splits each sucrose molecule into one glucose and one fructose molecule. It is sweeter than an equivalent sucrose solution by weight and is also more hygroscopic, so it can be used to make a product that stays moist longer compared to sucrose. This property is particularly valued by bakers and is found mainly in baked goods and confectionary as well as tobacco products. |
| 3 | --Inositol | No | Naturally occurring in various cereals, nuts, beans and fruit, inositol is a kind of sugar alcohol that is also classified as a B vitamin (often referred to as vitamin B8). |
| 3 | --Sucrose | Yes | Sucrose or table sugar is a disaccharide extracted from sugar cane or sugar beet. It is still the most widely used sweetener in the food and drinks industry, with key product categories including soft drinks, confectionary and baked goods. |
| * Included based on an assumed sugar/water ratio of 70/30; in the analyses, only the sugar content was included. Data sources: Euromonitor International (Passport database). | | | |

## Calculation of variables

We multiplied glucose/fructose syrup, glucose/corn syrup, and high fructose corn syrup by a factor of 0.7 to account for the water content of these. In order to derive the mean sales-weighted sugar content of soft drinks, we divided total annual sugar volume sold through soft drinks by the total annual sales volume of soft drinks.

## R-code of the statistical analyses

#set announcement date and country here

announcement_year **=** 2016

announcement_month **=** 3

intervention_country **=** "United Kingdom"

#calculate the first post intervention year (year before announcement, if announcement was in first half of the year; year of announcement, if announcement was in second half of the year)

post_year **=** ifelse**(**announcement_month**<**7, announcement_year**-**1, announcement_year**)**

###########################################################################

#load ingredients data and clean up

###########################################################################

ingredients **<-** read.csv**(**'C:/.../ingredients_09_22.csv'**)**

# Clearn data: change values to numbers

ingredients **<-** mutate**(**ingredients,

X2009**=**as.numeric**(**X2011**)**,

X2010**=**as.numeric**(**X2011**)**,

X2011**=**as.numeric**(**X2011**)**,

X2012**=**as.numeric**(**X2012**)**,

X2013**=**as.numeric**(**X2013**)**,

X2014**=**as.numeric**(**X2014**)**,

X2015**=**as.numeric**(**X2015**)**,

X2016**=**as.numeric**(**X2016**)**,

X2017**=**as.numeric**(**X2017**)**,

X2018**=**as.numeric**(**X2018**)**,

X2019**=**as.numeric**(**X2019**)**,

X2020**=**as.numeric**(**X2020**)**,

X2021**=**as.numeric**(**X2021**)**,

X2022**=**as.numeric**(**X2022**)**,

Country**=**as.factor**(**Country**)**,

Category**=**as.factor**(**Category**)**,

Categorization_Type**=**as.factor**(**Categorization_Type**)**,

Unit**=**as.factor**(**Unit**))**

#filter data: only tonnes, only sugar (according to our definition), no concentrates

ingredients **<-** ingredients %>%

filter**(**Unit **==** "Tonnes"**)** %>%

filter**(**Category **==**"Dextrose"**|**

Category**==**"Fructose"**|**

Category**==**"Sucrose"**|**

Category**==**"Invert Sugar"**|**

Category**==**"Glucose/Corn Syrup"**|**

Category**==**"Glucose/Fructose Syrup"**|**

Category**==**"High Fructose Corn Syrup"**)**%>%

filter**(**Categorization_Type **!=**"Concentrates"**)**

#move years to rows

ingredients_new **<-** pivot_longer**(**ingredients,col**=**5**:**18, names_to**=**"Year",values_to**=**"Total_Sugar"**)**

# get rid of X and define Year as integer

ingredients_new**$**Year **<-** gsub**(**"X","",ingredients_new**$**Year**)**

ingredients_new **<-** mutate**(**ingredients_new,Year**=**as.integer**(**Year**))**

#multiply by 0.7 for syrups

ingredients_new **<-** ingredients_new %>%

mutate**(**Sugar**=**ifelse**(**Category**==**"Glucose/Corn Syrup"**|**

Category**==**"Glucose/Fructose Syrup"**|**

Category**==**"High Fructose Corn Syrup",

Total_Sugar*****0.7,Total_Sugar**))**

#group by country

ingredients_grouped **<-** ingredients_new %>%

group_by**(**Country,Year**)** %>%

summarize**(**sugar_total**=**sum**(**Sugar**))**

###########################################################################

#load market data and clean up

###########################################################################

market **<-** read.csv**(**'C:/.../market_09_22.csv'**)**

#move years to rows

market_new **<-** pivot_longer**(**market,col**=**5**:**18, names_to**=**"Year",values_to**=**"Volume"**)**

# get rid of X and define Year as integer, and country as factor

market_new**$**Year **<-** gsub**(**"X","",market_new**$**Year**)**

market_new **<-** mutate**(**market_new,Year**=**as.integer**(**Year**)**, Country**=**as.factor**(**Country**))**

#group by country

market_grouped**<-** market_new %>%

group_by**(**Country,Year**)**%>%

summarize**(**volume_total**=**sum**(**Volume**)***1000000**)**

###########################################################################

#merge market data to ingredients data

###########################################################################

final_data_09_22 **<-** merge**(**ingredients_grouped,market_grouped,by.x **=** c**(**'Country','Year'**)**, by.y**=** c**(**'Country','Year'**))**

#calculate mean sales weighted sugar content

final_data_09_22 **<-** mutate**(**final_data_09_22, sugar_content **=** **(**sugar_total*****1000000**)/(**volume_total*****10**))**

#Filter for relevant countries

data_for_analysis **<-** final_data_09_22 %>% filter **(**

Country **==** intervention_country **|**

Country **==** "Austria" **|**

Country **==** "Germany" **|**

Country **==** "Netherlands" **|**

Country **==** "Denmark" **|**

Country **==** "Italy"**|**

Country **==** "Switzerland"**|**

Country **==** "Greece" **|**

Country **==** "Sweden"

**)**

#create year variable (year 1 is 2009)

its **<-** data_for_analysis %>%

mutate**(**year **=** Year**-**2008**)**

#create post and country variable

its **<-** its %>%

mutate**(**post **=**

ifelse **((**announcement_month**<**7 **&** Year**>=(**announcement_year**-**1**))**,1,

ifelse**((**announcement_month**>**6 **&** Year**>=(**announcement_year**))**,1,0**))**,

country**=**

ifelse **(**Country**==**intervention_country,1,0**))**

########################################################################

# SynthC ###############################################################

########################################################################

#create treat variable (1 for intervention country post-intervention, 0 for all else)

its**$**treat **<-** its**$**post ***** its**$**country

#run synthetic control model

sc **<-** gsynth**(**sugar_content **~** treat, X**=NULL**,

data **=** its, parallel**=FALSE**, na.rm**=**T,

index **=** c**(**"Country","year"**)**,r **=** c**(**1,2**)**,

CV **=** **TRUE**, se **=** **TRUE**, normalize **=** T,

inference **=** "parametric", nboots **=** 1000**)**

#display results of sc model

print**(**sc**)**

#plot results of sc

plot**(**sc, type **=** "counterfactual", raw **=** "none", main**=**""**)**

plot**(**sc, type **=** "gap", xlab **=** "Year", ylab**=**"Treatment Effect (g of sugar/100ml)",main **=** intervention_country**)**

plot**(**sc, type **=** "raw", main **=** intervention_country**)**

########################################################################

# cITS #################################################################

########################################################################

#take average of countries

#group by country

its_control_average**<-** its %>%

group_by**(**Year,country,year,post**)**%>%

summarize**(**sugar_content_average**=** mean**(**sugar_content**))** %>%

arrange**(**desc**(**country**))**

#set up data for cITS, create variables:

#intervention_year (counts years from 2009 only for intervention country)

#trend (in paper: year_post,starts to count after intervention)

#intervention_trend (in paper: year_post*country, starts to count after intervention, only for intervention country)

its_control_average**<-** mutate**(**its_control_average,

intervention_year**=**year*****country,

trend**=(**Year**-**post_year**+**1**)***post,

intervention_post**=**country*****post,

intervention_trend**=(**Year**-**post_year**+**1**)***post*****country**)**

#Fit the GLS regression model

model **<-** gls**(**sugar_content_average **~** year **+** country **+** intervention_year **+** post **+** trend **+** intervention_post **+** intervention_trend,

data**=**its_control_average,correlation**=NULL**,method**=**"ML"**)**

#Display results of regression and confidence intervals of estimates

summary**(**model**)**

intervals**(**model**)**

#plot results of cITS

plot**(**its_control_average**$**year**[**1**:**14**]**,its_control_average**$**sugar_content_average**[**1**:**14**]**,

ylim**=**c**(**3.5,8**)**,

ylab**=**"average sugar content in g/100ml",

xlab**=**"Year",

pch**=**20,

col**=**"lightblue",xaxt**=**"n",main**=**intervention_country**)**

# Add x-axis year labels

axis**(**1, at**=**1**:**14, labels**=**its_control_average**$**Year**[**1**:**14**])**

# Label the policy change

abline**(**v**=**post_year**-**2008.5,lty**=**2**)**

points**(**its_control_average**$**year**[**15**:**28**]**,

its_control_average**$**sugar_content_average**[**15**:**28**]**,

col**=**"pink",

pch**=**20**)**

# Plot the first line segment for the intervention group

lines**(**its_control_average**$**year**[**1**:(**post_year**-**2009**)]**, fitted**(**model**)[**1**:(**post_year**-**2009**)]**, col**=**"blue",lwd**=**1**)**

# Add the second line segment for the intervention group

lines**(**its_control_average**$**year**[(**post_year**-**2008**):**14**]**, fitted**(**model**)[(**post_year**-**2008**):**14**]**, col**=**"blue",lwd**=**1**)**

# Plot the first line segment for the control group

lines**(**its_control_average**$**year**[**15**:(**post_year**-**1995**)]**, fitted**(**model**)[**15**:(**post_year**-**1995**)]**, col**=**"red",lwd**=**1**)**

# Add the second line segment for the control

lines**(**its_control_average**$**year**[(**post_year**-**1994**):**28**]**, fitted**(**model**)[(**post_year**-**1994**):**28**]**, col**=**"red",lwd**=**1**)**

# Additional results

## Full results and further graphics of SC analysis

eFigure 2 depicts the observed outcome (mean sales-weighted sugar content) and the modeled synthetic control.


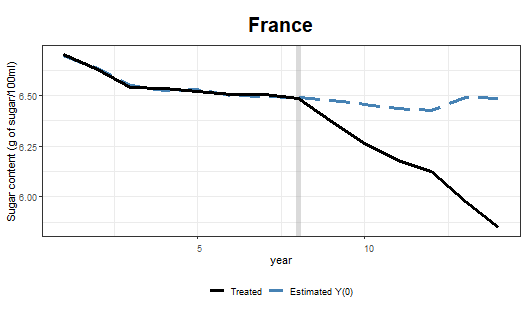

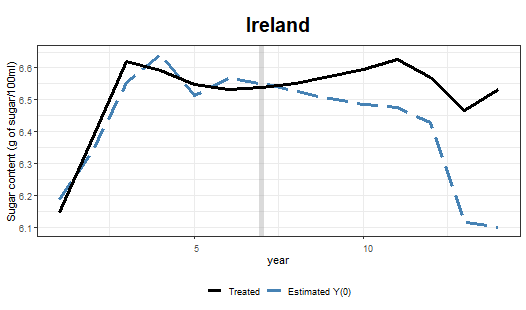


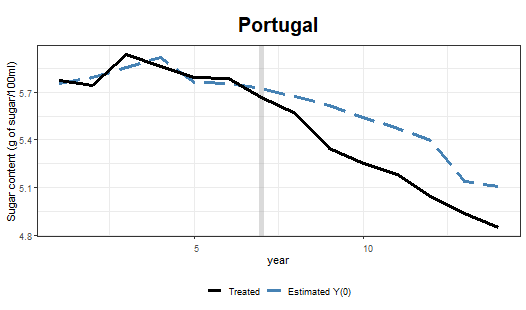

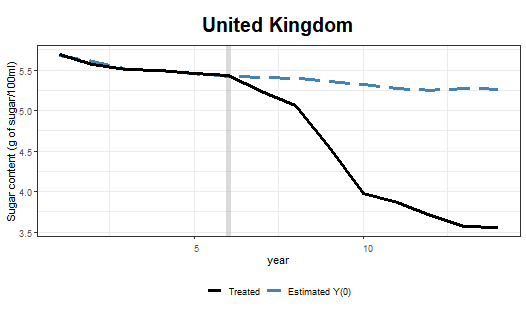


***eFigure 2: Graphical representation of observed outcome (black) and modeled synthetic control (dashed blue).*** *Notes: The vertical grey line denotes the intervention time in our study design.*

| ***eTable 5: Full Results of SC analysis*** | | | | | |
| --- | --- | --- | --- | --- | --- |
| **France** | | | | | |
| Period | TE | S.E. | CI.lower | CI.upper | p.value |
| -7 | 0.005838 | 0.03628 | -0.06528 | 0.07695 | 0.8722 |
| -6 | -0.004282 | 0.02838 | -0.05991 | 0.05134 | 0.8801 |
| -5 | -0.009721 | 0.06561 | -0.13832 | 0.11888 | 0.8822 |
| -4 | 0.008175 | 0.07764 | -0.14399 | 0.16034 | 0.9161 |
| -3 | -0.010235 | 0.06055 | -0.12892 | 0.10845 | 0.8658 |
| -2 | 0.002313 | 0.02768 | -0.05194 | 0.05656 | 0.9334 |
| -1 | 0.010778 | 0.02313 | -0.03456 | 0.05611 | 0.6413 |
| 0 | -0.002865 | 0.04052 | -0.08228 | 0.07655 | 0.9436 |
| 1 | -0.103429 | 0.08778 | -0.27547 | 0.06861 | 0.2387 |
| 2 | -0.194569 | 0.13282 | -0.45488 | 0.06574 | 0.1429 |
| 3 | -0.253849 | 0.19546 | -0.63694 | 0.12925 | 0.194 |
| 4 | -0.299108 | 0.28254 | -0.85287 | 0.25465 | 0.2898 |
| 5 | -0.512604 | 0.61213 | -1.71235 | 0.68714 | 0.4024 |
| 6 | -0.637016 | 0.52784 | -1.67157 | 0.39754 | 0.2275 |
| ATE | -0.3334 | 0.2845 | -0.8909 | 0.2241 | 0.2411 |
| **Ireland** | | | | | |
| Period | TE | S.E. | CI.lower | CI.upper | p.value |
| -6 | -0.04166 | 0.05855 | -0.15642 | 0.0731 | 0.4768 |
| -5 | 0.04114 | 0.03991 | -0.03709 | 0.11937 | 0.3027 |
| -4 | 0.06437 | 0.10467 | -0.14078 | 0.26952 | 0.5386 |
| -3 | -0.05259 | 0.10383 | -0.25609 | 0.15092 | 0.6125 |
| -2 | 0.03401 | 0.06945 | -0.10211 | 0.17014 | 0.6243 |
| -1 | -0.03379 | 0.03154 | -0.09561 | 0.02803 | 0.284 |
| 0 | -0.01149 | 0.05785 | -0.12488 | 0.10189 | 0.8425 |
| 1 | 0.02554 | 0.1102 | -0.19045 | 0.24152 | 0.8168 |
| 2 | 0.06884 | 0.16384 | -0.25227 | 0.38995 | 0.6744 |
| 3 | 0.10835 | 0.24301 | -0.36795 | 0.58465 | 0.6557 |
| 4 | 0.15052 | 0.3179 | -0.47256 | 0.7736 | 0.6359 |
| 5 | 0.14086 | 0.40926 | -0.66128 | 0.94301 | 0.7307 |
| 6 | 0.35463 | 0.70926 | -1.03549 | 1.74476 | 0.6171 |
| 7 | 0.43458 | 0.65154 | -0.84242 | 1.71158 | 0.5048 |
| ATE | 0.1833 | 0.3359 | -0.475 | 0.8416 | 0.5852 |
| **Portugal** | | | | | |
| Period | TE | S.E. | CI.lower | CI.upper | p.value |
| -6 | 0.01404 | 0.04821 | -0.08046 | 0.10853 | 0.77096 |
| -5 | -0.0501 | 0.03395 | -0.11664 | 0.01644 | 0.14004 |
| -4 | 0.08272 | 0.09711 | -0.10762 | 0.27306 | 0.39431 |
| -3 | -0.05439 | 0.09501 | -0.24061 | 0.13182 | 0.56699 |
| -2 | 0.03404 | 0.06549 | -0.09431 | 0.1624 | 0.60317 |
| -1 | 0.02799 | 0.0328 | -0.0363 | 0.09229 | 0.39345 |
| 0 | -0.05431 | 0.05667 | -0.16537 | 0.05676 | 0.33787 |
| 1 | -0.10978 | 0.10545 | -0.31645 | 0.09689 | 0.29783 |
| 2 | -0.27117 | 0.15735 | -0.57956 | 0.03723 | 0.08482 |
| 3 | -0.2855 | 0.23214 | -0.74049 | 0.16948 | 0.21874 |
| 4 | -0.28933 | 0.30742 | -0.89186 | 0.31321 | 0.34663 |
| 5 | -0.34995 | 0.40227 | -1.13838 | 0.43848 | 0.38433 |
| 6 | -0.20191 | 0.70402 | -1.58177 | 1.17795 | 0.77427 |
| 7 | -0.26221 | 0.63538 | -1.50754 | 0.98312 | 0.67984 |
| ATE | -0.2528 | 0.3316 | -0.9027 | 0.3971 | 0.4458 |
| **UK** | | | | | |
| Period | TE | S.E. | CI.lower | CI.upper | p.value |
| -5 | 0.020377 | 0.02515 | -0.02891 | 0.06966 | 4.18E-01 |
| -4 | -0.030277 | 0.02839 | -0.08592 | 0.02537 | 2.86E-01 |
| -3 | -0.003206 | 0.0739 | -0.14805 | 0.14164 | 9.65E-01 |
| -2 | 0.00842 | 0.06577 | -0.12049 | 0.13733 | 8.98E-01 |
| -1 | -0.002512 | 0.04235 | -0.08551 | 0.08049 | 9.53E-01 |
| 0 | 0.007197 | 0.04315 | -0.07738 | 0.09177 | 8.68E-01 |
| 1 | **-0.172956** | 0.07232 | -0.3147 | -0.03122 | 1.68E-02 |
| 2 | **-0.32489** | 0.11604 | -0.55232 | -0.09746 | 5.11E-03 |
| 3 | **-0.823019** | 0.1555 | -1.12779 | -0.51825 | 1.20E-07 |
| 4 | **-1.338363** | 0.22239 | -1.77424 | -0.90249 | 1.77E-09 |
| 5 | **-1.406923** | 0.28163 | -1.9589 | -0.85494 | 5.86E-07 |
| 6 | **-1.543963** | 0.35173 | -2.23334 | -0.85458 | 1.14E-05 |
| 7 | **-1.706276** | 0.53729 | -2.75934 | -0.65321 | 1.50E-03 |
| 8 | **-1.711533** | 0.45591 | -2.60511 | -0.81796 | 1.74E-04 |
| ATE | **-1.128** | 0.2409 | -1.601 | -0.6564 | 2.80E-06 |
| *Bold: denotes statistical significance at an alpha level of 5%. Abbreviations: CS: Synthetic control; ATE: Average treatment effect (across all post-intervention periods); TE: treatment effect (for each time period post-intervention); S.E.: Standard error; CI: Confidence interval. 0 denotes the last pre-intervention period.* | | | | | |

## Results and graphics of cITS

Results of the secondary analysis are summarized in eTable 6, which provides the estimates for the level and trend changes as well as their respective confidence intervals and p-values.

| ***eTable 6: Results of controlled interrupted time series analysis of impact of tiered soft drink taxes on sales-weighted sugar content*** | | | |
| --- | --- | --- | --- |
|  | ***France*** |  |  |
|  | *Coefficient* | *95%-CI* | *p-value* |
| country*post (level change) | 0.02 | [-0.08; 0.12] | 0.68 |
| country*year_post (trend change) | **-0.03** | [-0.06; -0.01] | 0.01 |
|  | ***Ireland*** |  |  |
|  | *Coefficient* | *95%-CI* | *p-value* |
| country*post (level change) | -0.05 | [-0.29; 0.19] | 0.67 |
| country*year_post (trend change) | -0.02 | [-0.08; 0.04] | 0.48 |
|  | ***Portugal*** |  |  |
|  | *Coefficient* | *95%-CI* | *p-value* |
| country*post (level change) | -0.15 | [-0.32; 0.01] | 0.07 |
| country*year_post (trend change) | **-0.06** | [-0.10; -0.02] | 0.0060 |
|  | ***UK*** |  |  |
|  | *Coefficient* | *95%-CI* | *p-value* |
| country*post (level change) | -0.08 | [-0.47; 0.32] | 0.70 |
| country*year_post (trend change) | **-0.17** | [-0.28; -0.07] | 0.003 |
| *Shown is the impact of tiered soft drink taxes on sales-weighted sugar content. Bold: denotes statistical significance at an alpha level of 5 %.* | | | |

The results of the cITS revealed a negative trend change in all four intervention countries. In line with the main analysis, this effect was largest in the UK, where the trend change post-intervention amounted to ‑0.17 g/100 ml per year (95%-CI = [-0.28; -0.07]), with effects of a smaller magnitude in Portugal (-0.06 g/100 ml per year, 95%-CI = [‑0.10; ‑0.02]) and France (-0.03 g/100 ml per year, 95%-CI = [‑0.06; ‑0.01]). Ireland showed a comparatively smaller trend change of ‑0.02 g/100 ml per year (95%-CI = [-0.08; 0.04]).

The level changes showed an immediate decrease in sugar content after the invention in Portugal (‑0.15 g/100 ml, 95%-CI = [-0.32;0.01]), the UK (-0.08 g/100 ml, 95%-CI=[-0.47; 0.32]) and Ireland (-0.05 g/100 ml, 95%-CI = [-0.29; 0.19]), whereas France showed a small but positive initial level change after the intervention (0.02 g/100 ml, 95%-CI = [-0.08; 0.12]).

As indicated in eTable 6, significant effects were only observed for the trend changes in France, Portugal and the UK. In Ireland, the confidence interval of the trend change crossed the null, as did the confidence intervals of the level changes of all four countries. The parallel trends assumption was violated only for Ireland, see eFigure 4. A more elaborate table of results and a graphical representation of the secondary analysis can be found in eTable 7 and eFigure 3, respectively.

| ***eTable 7: Full Results of cITS analysis*** | | | | | |
| --- | --- | --- | --- | --- | --- |
| **France** |  |  |  |  |  |
|  | Value | Std.Error | lower | upper | p-value |
| Intercept | **6.079149** | 0.02376635 | 6.02957299 | 6.12872448 | 0 |
| year | **-0.026401** | 0.00470644 | -0.0362187 | -0.0165838 | 0 |
| country | **0.593651** | 0.0336107 | 0.52353986 | 0.66376124 | 0 |
| year*country | -0.000429 | 0.00665591 | -0.0143133 | 0.01345464 | 0.9492 |
| post | 0.002372 | 0.03455309 | -0.0697041 | 0.07444887 | 0.9459 |
| year_post | **-0.04026** | 0.00867825 | -0.058362 | -0.022157 | 0.0002 |
| country*post | 0.020555 | 0.04886544 | -0.0813766 | 0.12248643 | 0.6785 |
| country*year_post | **-0.034211** | 0.01227289 | -0.0598115 | -0.0086098 | 0.0114 |
| **Ireland** |  |  |  |  |  |
|  | Value | Std.Error | lower | upper | p-value |
| Intercept | **6.070989** | 0.0629014 | 5.93977909 | 6.20219914 | 0 |
| year | -0.023681 | 0.01406518 | -0.0530208 | 0.00565808 | 0.1078 |
| country | **0.205587** | 0.08895602 | 0.02002809 | 0.39114609 | 0.0316 |
| year*country | **0.074325** | 0.01989117 | 0.03283264 | 0.11581714 | 0.0013 |
| post | 0.019517 | 0.08079831 | -0.1490251 | 0.18805961 | 0.8116 |
| year_post | -0.040685 | 0.01989117 | -0.0821776 | 0.00080694 | 0.0542 |
| country*post | -0.04948 | 0.11426607 | -0.2878346 | 0.18887506 | 0.6696 |
| country*year_post | -0.020419 | 0.02813036 | -0.079098 | 0.03825977 | 0.4763 |
| **Portugal** |  |  |  |  |  |
|  | Value | Std.Error | lower | upper | p-value |
| Intercept | **6.070989** | 0.04346785 | 5.98031677 | 6.16166146 | 0 |
| year | **-0.023681** | 0.00971971 | -0.0439563 | -0.0034064 | 0.0243 |
| country | **-0.225809** | 0.06147282 | -0.3540389 | -0.0975787 | 0.0015 |
| year*country | 0.010625 | 0.01374574 | -0.0180482 | 0.03929808 | 0.4486 |
| post | 0.019517 | 0.05583546 | -0.0969535 | 0.13598802 | 0.7303 |
| year_post | **-0.040685** | 0.01374574 | -0.0693584 | -0.0120122 | 0.0077 |
| country*post | -0.151521 | 0.07896327 | -0.3162354 | 0.01319363 | 0.0694 |
| country*year_post | **-0.059438** | 0.01943941 | -0.0999881 | -0.0188883 | 0.0062 |
| **UK** |  |  |  |  |  |
|  | Value | Std.Error | lower | upper | p-value |
| Intercept | **6.063057** | 0.11738695 | 5.81819242 | 6.3079222 | 0 |
| year | -0.020707 | 0.03014219 | -0.0835825 | 0.04216855 | 0.5 |
| country | **-0.370352** | 0.16601022 | -0.7166437 | -0.0240612 | 0.0373 |
| year*country | -0.026998 | 0.04262749 | -0.115917 | 0.06192182 | 0.5337 |
| post | 0.033093 | 0.13409604 | -0.2466263 | 0.31281256 | 0.8076 |
| year_post | -0.040794 | 0.03587638 | -0.1156308 | 0.03404286 | 0.2689 |
| country*post | -0.077426 | 0.18964043 | -0.4730093 | 0.31815678 | 0.6874 |
| country*year_post | **-0.172128** | 0.05073686 | -0.2779631 | -0.0662926 | 0.0029 |
| *Bold: denotes statistical significance at an alpha level of 5%. Abbreviations: CS: Synthetic control; ATE: Average treatment effect (across all post-intervention periods); TE: treatment effect (for each time period post-intervention); S.E.: Standard error; CI: Confidence interval. 0 denotes the last pre-intervention period.* | | | | | |


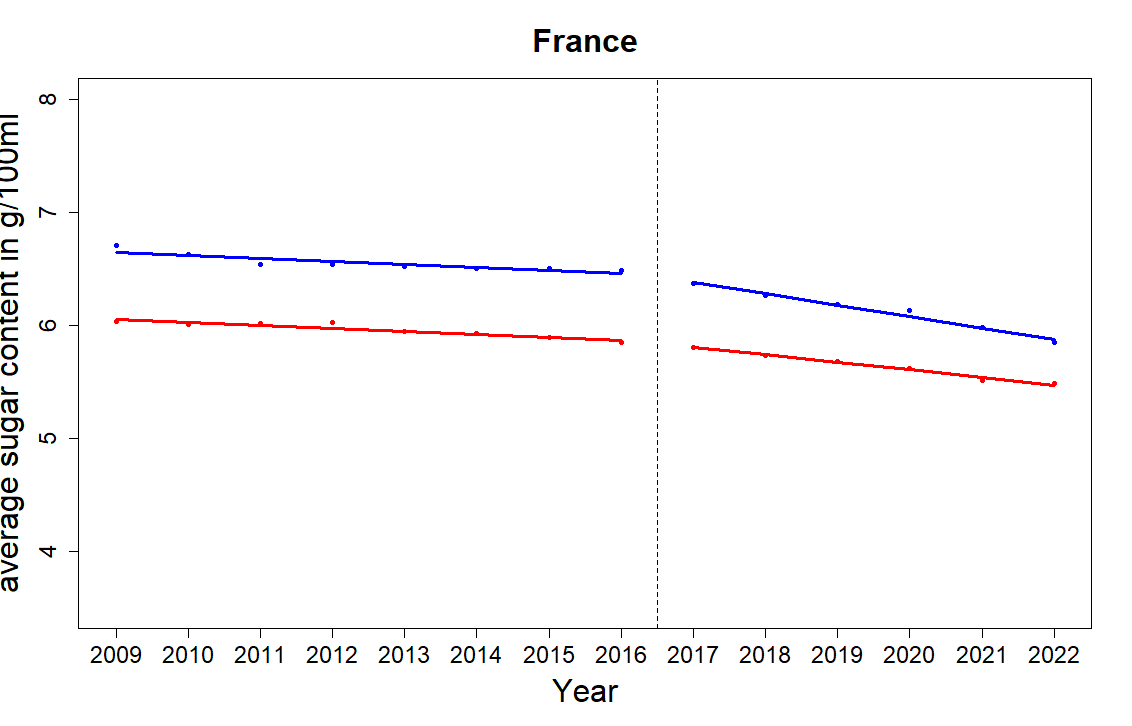

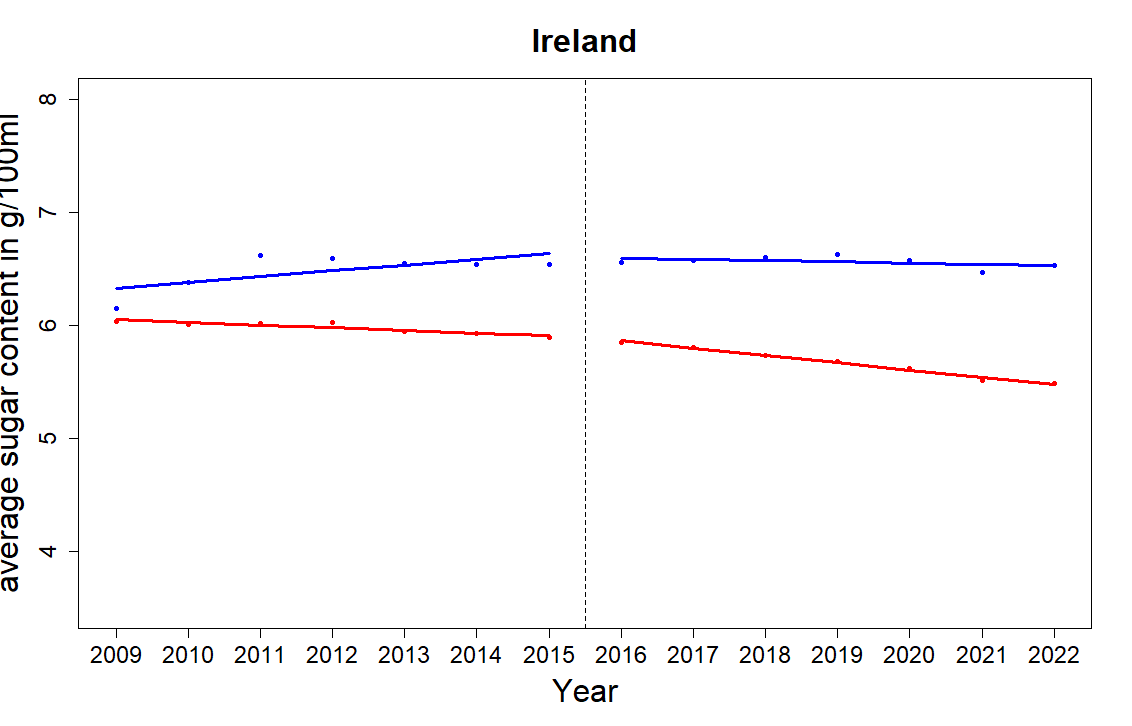


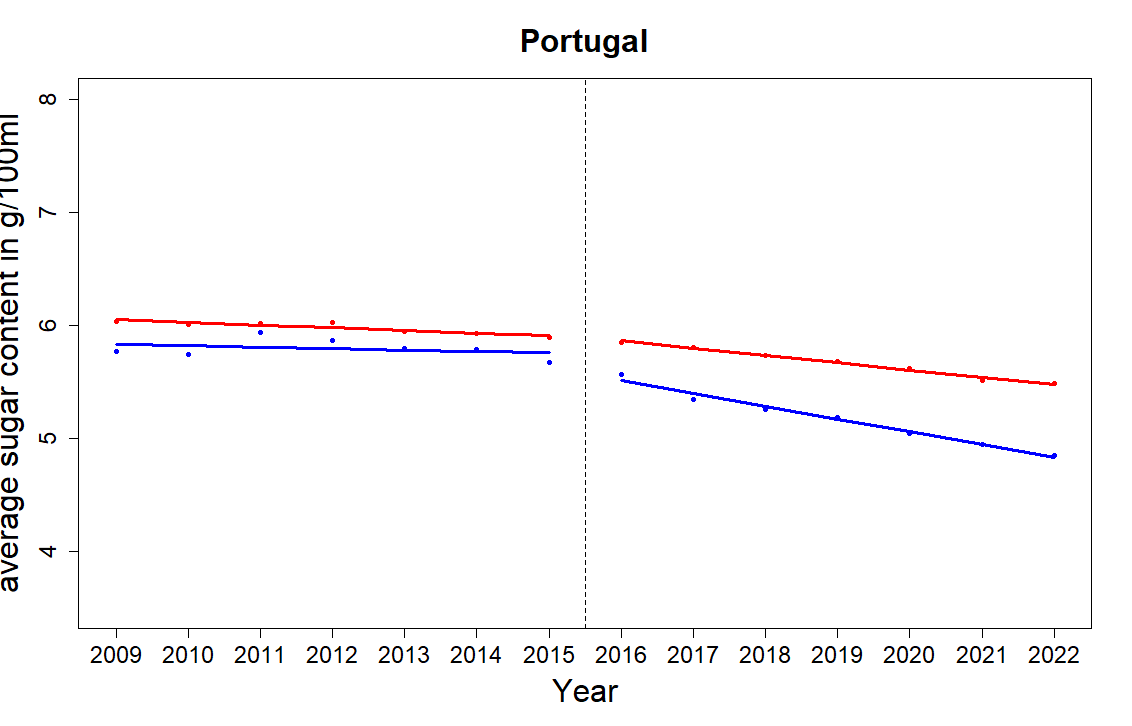

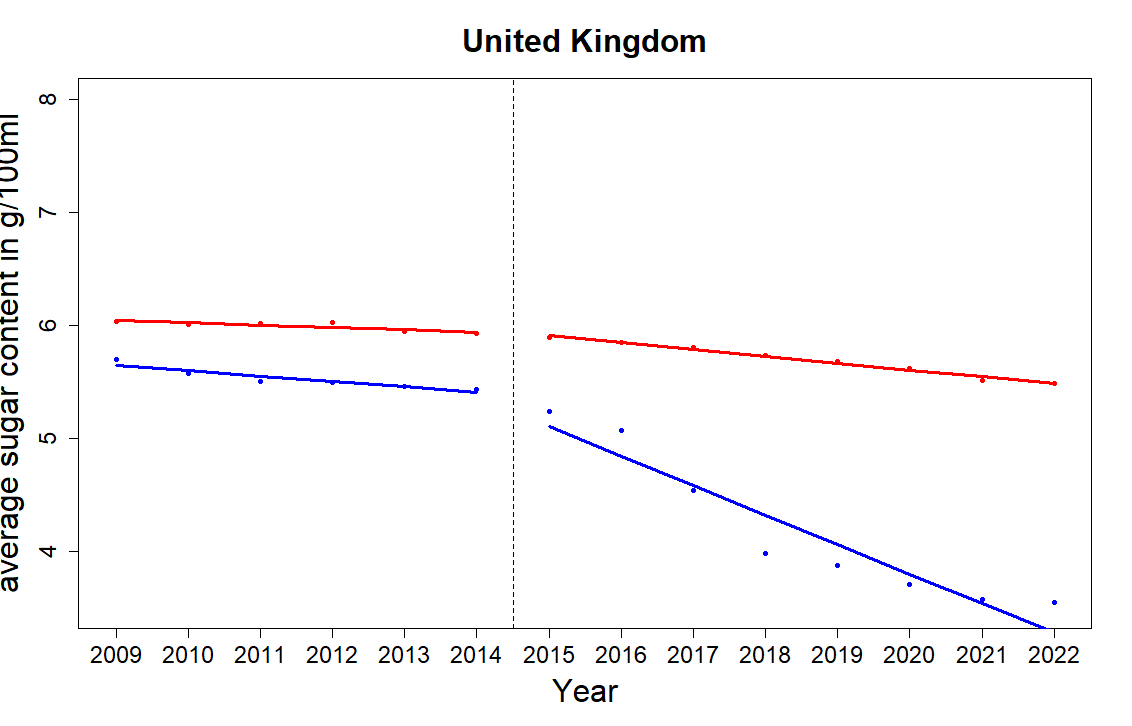


***eFigure 3: Results of controlled interrupted time series.*** *Dots denote observed data, lines represent the regression lines. Red are the average of the control countries; blue are the respective intervention countries. The horizontal line represents the time of the intervention as defined in this study.*

## Sensitivity analyses

| ***eTable 8: Sensitivity analysis of SC and cITS, setting the intervention date one year later*** | | | | | |
| --- | --- | --- | --- | --- | --- |
| **France** | | | | | |
| Period | TE | S.E. | CI.lower | CI.upper | p.value |
| 1 | -0.104019 | 0.12926 | -0.35736 | 0.14932 | 0.421 |
| 2 | -0.138988 | 0.16845 | -0.46914 | 0.19116 | 0.4093 |
| 3 | -0.154255 | 0.20085 | -0.54791 | 0.2394 | 0.4425 |
| 4 | -0.267371 | 0.39948 | -1.05033 | 0.51559 | 0.5033 |
| 5 | -0.379882 | 0.43464 | -1.23177 | 0.472 | 0.3821 |
| ATE | -0.2089 | 0.2204 | -0.6408 | 0.223 | 0.3432 |
| country*post (level change) | -0.004659 | 0.05664087 | -0.1228098 | 0.11349177 | 0.9353 |
| year*country*post (trend change) | **-0.033906** | 0.01591471 | -0.0671038 | -0.0007088 | 0.0457 |
| **Ireland** | | | | | |
| Period | TE | S.E. | CI.lower | CI.upper | p.value |
| 1 | 0.05309 | 0.11504 | -0.17238 | 0.27856 | 0.6444 |
| 2 | 0.08718 | 0.17533 | -0.25647 | 0.43083 | 0.619 |
| 3 | 0.12451 | 0.24475 | -0.3552 | 0.60422 | 0.611 |
| 4 | 0.10963 | 0.34033 | -0.5574 | 0.77667 | 0.7473 |
| 5 | 0.30983 | 0.69969 | -1.06154 | 1.6812 | 0.6579 |
| 6 | 0.38765 | 0.6487 | -0.88378 | 1.65909 | 0.5501 |
| ATE | 0.1786 | 0.3432 | -0.494 | 0.8513 | 0.6027 |
| country*post (level change) | -0.003832 | 0.12316 | -0.260739 | 0.25307548 | 0.9755 |
| year*country*post (trend change) | -0.017953 | 0.03093248 | -0.0824767 | 0.04657139 | 0.5681 |
| **Portugal** | | | | | |
| Period | TE | S.E. | CI.lower | CI.upper | p.value |
| 1 | -0.20346 | 0.11993 | -0.438509 | 0.03159 | 0.08978 |
| 2 | -0.194487 | 0.18184 | -0.550882 | 0.16191 | 0.28482 |
| 3 | -0.177489 | 0.26823 | -0.703203 | 0.34822 | 0.50815 |
| 4 | -0.2157 | 0.39285 | -0.985669 | 0.55427 | 0.58296 |
| 5 | -0.009306 | 0.82494 | -1.626152 | 1.60754 | 0.991 |
| 6 | -0.060457 | 0.70849 | -1.449078 | 1.32816 | 0.932 |
| ATE | -0.1435 | 0.3894 | -0.9066 | 0.6196 | 0.7125 |
| country*post (level change) | -0.209682 | 0.0916908 | -0.4009456 | -0.0184183 | 0.0332 |
| year*country*post (trend change) | -0.033984 | 0.02302878 | -0.0820207 | 0.01405366 | 0.1556 |
| **UK** | | | | | |
| Period | TE | S.E. | CI.lower | CI.upper | p.value |
| 1 | -0.218494 | 0.1389 | -0.49074 | 0.05375 | 1.16E-01 |
| 2 | **-0.685024** | 0.18252 | -1.04275 | -0.3273 | 1.75E-04 |
| 3 | **-1.15731** | 0.27513 | -1.69656 | -0.61806 | 2.60E-05 |
| 4 | **-1.187125** | 0.34251 | -1.85843 | -0.51582 | 5.28E-04 |
| 5 | **-1.284305** | 0.39752 | -2.06343 | -0.50518 | 1.23E-03 |
| 6 | **-1.353576** | 0.53134 | -2.39499 | -0.31217 | 1.09E-02 |
| 7 | **-1.342386** | 0.56175 | -2.4434 | -0.24137 | 1.69E-02 |
| ATE | -1.033 | 0.3175 | -1.655 | -0.4103 | 0.001146 |
| country*post (level change) | -0.314666 | 0.18657868 | -0.703862 | 0.0745306 | 0.1072 |
| year*country*post (trend change) | **-0.139906** | 0.0459325 | -0.2357193 | -0.0440923 | 0.0064 |
| *Bold: denotes statistical significance at an alpha level of 5%. Abbreviations: CS: Synthetic control; cITS: Controlled interrupted times series; ATE: Average treatment effect (across all post-intervention periods); TE: treatment effect (for each time period post-intervention); S.E.: Standard error; CI: Confidence interval* | | | | | |

| ***eTable 9: Sensitivity analysis of SC method, leaving one of the control countries out at a time*** | | | | | |
| --- | --- | --- | --- | --- | --- |
| **Intervention Country:** | **France** |  |  |  |  |
| **Leaving out:** | **Austria** |  |  |  |  |
| Post-Intervention period | TE | S.E. | CI.lower | CI.upper | p.value |
| 1 | -0.103548 | 0.08011 | -0.26057 | 0.05347 | 0.1962 |
| 2 | -0.196232 | 0.12839 | -0.44786 | 0.0554 | 0.1264 |
| 3 | -0.253454 | 0.16384 | -0.57457 | 0.06767 | 0.1219 |
| 4 | -0.293301 | 0.18038 | -0.64683 | 0.06023 | 0.1039 |
| 5 | -0.479983 | 0.35977 | -1.18513 | 0.22516 | 0.1822 |
| 6 | -0.609162 | 0.41089 | -1.41448 | 0.19616 | 0.1382 |
| ATE | -0.3226 | 0.1936 | -0.7021 | 0.05685 | 0.09565 |
| **Intervention Country:** | **France** |  |  |  |  |
| **Leaving out:** | **Germany** |  |  |  |  |
| Post-Intervention period | TE | S.E. | CI.lower | CI.upper | p.value |
| 1 | -0.10323 | 0.10281 | -0.30474 | 0.09828 | 0.3153 |
| 2 | -0.193768 | 0.15772 | -0.50289 | 0.11535 | 0.2192 |
| 3 | -0.252429 | 0.23605 | -0.71507 | 0.21022 | 0.2849 |
| 4 | -0.299685 | 0.35513 | -0.99572 | 0.39635 | 0.3987 |
| 5 | -0.512353 | 0.73422 | -1.9514 | 0.9267 | 0.4853 |
| 6 | -0.641331 | 0.64178 | -1.8992 | 0.61654 | 0.3177 |
| ATE | -0.3338 | 0.3477 | -1.015 | 0.3477 | 0.3371 |
| **Intervention Country:** | **France** |  |  |  |  |
| **Leaving out:** | **Netherlands** |  |  |  |  |
| Post-Intervention period | TE | S.E. | CI.lower | CI.upper | p.value |
| 1 | -0.110322 | 0.11217 | -0.33018 | 0.10953 | 0.3254 |
| 2 | -0.206851 | 0.16101 | -0.52242 | 0.10872 | 0.1989 |
| 3 | -0.2678 | 0.25777 | -0.77302 | 0.23742 | 0.2988 |
| 4 | -0.309022 | 0.4008 | -1.09457 | 0.47653 | 0.4407 |
| 5 | -0.503879 | 0.85311 | -2.17594 | 1.16818 | 0.5548 |
| 6 | -0.619791 | 0.72687 | -2.04442 | 0.80484 | 0.3938 |
| ATE | -0.3363 | 0.4029 | -1.126 | 0.4534 | 0.4039 |
| **Intervention Country:** | **France** |  |  |  |  |
| **Leaving out:** | **Denmark** |  |  |  |  |
| Post-Intervention period | TE | S.E. | CI.lower | CI.upper | p.value |
| 1 | -0.1060504 | 0.13392 | -0.36853 | 0.15643 | 0.4284 |
| 2 | -0.2010313 | 0.19545 | -0.5841 | 0.18204 | 0.3037 |
| 3 | -0.2609663 | 0.31979 | -0.88775 | 0.36582 | 0.4145 |
| 4 | -0.3020758 | 0.49816 | -1.27845 | 0.6743 | 0.5443 |
| 5 | -0.4640601 | 1.05165 | -2.52527 | 1.59715 | 0.659 |
| 6 | -0.5828235 | 0.85784 | -2.26416 | 1.09851 | 0.4969 |
| ATE | -0.3195 | 0.4973 | -1.294 | 0.6552 | 0.5206 |
| **Intervention Country:** | **France** |  |  |  |  |
| **Leaving out:** | **Italy** |  |  |  |  |
| Post-Intervention period | TE | S.E. | CI.lower | CI.upper | p.value |
| 1 | -0.101466 | 0.07916 | -0.25661 | 0.05368 | 0.1999 |
| 2 | -0.191341 | 0.12466 | -0.43567 | 0.05299 | 0.1248 |
| 3 | -0.249888 | 0.20399 | -0.6497 | 0.14992 | 0.2206 |
| 4 | -0.294834 | 0.32204 | -0.92601 | 0.33635 | 0.3599 |
| 5 | -0.511052 | 0.72316 | -1.92842 | 0.90631 | 0.4798 |
| 6 | -0.635322 | 0.57284 | -1.75806 | 0.48742 | 0.2674 |
| ATE | -0.3307 | 0.3119 | -0.942 | 0.2807 | 0.2891 |
| **Intervention Country:** | **France** |  |  |  |  |
| **Leaving out:** | **Switzerland** |  |  |  |  |
| Post-Intervention period | TE | S.E. | CI.lower | CI.upper | p.value |
| 1 | -0.103086 | 0.10521 | -0.3093 | 0.10313 | 0.3272 |
| 2 | -0.192559 | 0.15558 | -0.4975 | 0.11238 | 0.2158 |
| 3 | -0.249628 | 0.21379 | -0.66865 | 0.1694 | 0.243 |
| 4 | -0.293311 | 0.30161 | -0.88445 | 0.29783 | 0.3308 |
| 5 | -0.503828 | 0.6438 | -1.76565 | 0.75799 | 0.4339 |
| 6 | -0.627719 | 0.58586 | -1.77598 | 0.52054 | 0.284 |
| ATE | -0.3284 | 0.3113 | -0.9386 | 0.2819 | 0.2916 |
| **Intervention Country:** | **France** |  |  |  |  |
| **Leaving out:** | **Greece** |  |  |  |  |
| Post-Intervention period | TE | S.E. | CI.lower | CI.upper | p.value |
| 1 | -0.05873 | 0.0586 | -0.17358 | 0.05611 | 0.3162 |
| 2 | -0.12511 | 0.09868 | -0.31852 | 0.06829 | 0.2048 |
| 3 | -0.16418 | 0.14154 | -0.44159 | 0.11324 | 0.2461 |
| 4 | -0.1824 | 0.17677 | -0.52886 | 0.16406 | 0.3021 |
| 5 | -0.29809 | 0.41375 | -1.10903 | 0.51284 | 0.4712 |
| 6 | -0.4121 | 0.44251 | -1.27941 | 0.45521 | 0.3517 |
| ATE | -0.2068 | 0.1825 | -0.5644 | 0.1509 | 0.2572 |
| **Intervention Country:** | **France** |  |  |  |  |
| **Leaving out:** | **Sweden** |  |  |  |  |
| Post-Intervention period | TE | S.E. | CI.lower | CI.upper | p.value |
| 1 | -0.0989156 | 0.11001 | -0.31452 | 0.11669 | 0.3686 |
| 2 | -0.1896714 | 0.16909 | -0.52108 | 0.14174 | 0.262 |
| 3 | -0.2500589 | 0.24653 | -0.73325 | 0.23314 | 0.3104 |
| 4 | -0.2960543 | 0.33779 | -0.9581 | 0.36599 | 0.3808 |
| 5 | -0.4867614 | 0.68262 | -1.82468 | 0.85116 | 0.4758 |
| 6 | -0.6105439 | 0.59749 | -1.7816 | 0.56051 | 0.3069 |
| ATE | -0.322 | 0.3376 | -0.9836 | 0.3396 | 0.3401 |
| **Intervention Country:** | **Ireland** |  |  |  |  |
| **Leaving out:** | **Austria** |  |  |  |  |
| Post-Intervention period | TE | S.E. | CI.lower | CI.upper | p.value |
| 1 | 0.04104 | 0.11339 | -0.18121 | 0.26329 | 0.7174 |
| 2 | 0.079564 | 0.15752 | -0.22916 | 0.38829 | 0.6135 |
| 3 | 0.129073 | 0.23545 | -0.33239 | 0.59054 | 0.5836 |
| 4 | 0.165811 | 0.28627 | -0.39527 | 0.72689 | 0.5624 |
| 5 | 0.137242 | 0.32568 | -0.50108 | 0.77557 | 0.6735 |
| 6 | 0.251844 | 0.47259 | -0.67442 | 1.1781 | 0.5941 |
| 7 | 0.35398 | 0.54298 | -0.71024 | 1.4182 | 0.5145 |
| ATE | 0.1655 | 0.2691 | -0.3618 | 0.6928 | 0.5385 |
| **Intervention Country:** | **Ireland** |  |  |  |  |
| **Leaving out:** | **Germany** |  |  |  |  |
| Post-Intervention period | TE | S.E. | CI.lower | CI.upper | p.value |
| 1 | 0.02571 | 0.11808 | -0.20571 | 0.25713 | 0.8276 |
| 2 | 0.068 | 0.18281 | -0.2903 | 0.42629 | 0.7099 |
| 3 | 0.10567 | 0.27052 | -0.42455 | 0.63589 | 0.6961 |
| 4 | 0.1461 | 0.36646 | -0.57215 | 0.86435 | 0.6901 |
| 5 | 0.14147 | 0.49657 | -0.83179 | 1.11473 | 0.7757 |
| 6 | 0.35385 | 0.88717 | -1.38497 | 2.09266 | 0.69 |
| 7 | 0.44562 | 0.78258 | -1.08821 | 1.97945 | 0.5691 |
| ATE | 0.1838 | 0.4069 | -0.6137 | 0.9813 | 0.6515 |
| **Intervention Country:** | **Ireland** |  |  |  |  |
| **Leaving out:** | **Netherlands** |  |  |  |  |
| Post-Intervention period | TE | S.E. | CI.lower | CI.upper | p.value |
| 1 | 0.036064 | 0.11562 | -0.19056 | 0.26268 | 0.7551 |
| 2 | 0.083 | 0.16769 | -0.24567 | 0.41167 | 0.6206 |
| 3 | 0.13175 | 0.2357 | -0.33022 | 0.59372 | 0.5762 |
| 4 | 0.17803 | 0.331 | -0.47072 | 0.82678 | 0.5907 |
| 5 | 0.165842 | 0.47683 | -0.76873 | 1.10042 | 0.728 |
| 6 | 0.345484 | 0.91096 | -1.43997 | 2.13094 | 0.7045 |
| 7 | 0.417112 | 0.79777 | -1.14648 | 1.9807 | 0.6011 |
| ATE | 0.1939 | 0.4006 | -0.5913 | 0.9791 | 0.6284 |
| **Intervention Country:** | **Ireland** |  |  |  |  |
| **Leaving out:** | **Denmark** |  |  |  |  |
| Post-Intervention period | TE | S.E. | CI.lower | CI.upper | p.value |
| 1 | 0.0826 | 0.06348 | -0.041819 | 0.20701 | 0.1932 |
| 2 | 0.12383 | 0.15301 | -0.176061 | 0.42373 | 0.41833 |
| 3 | 0.19975 | 0.21527 | -0.222175 | 0.62167 | 0.35346 |
| 4 | 0.26088 | 0.33839 | -0.402344 | 0.9241 | 0.44073 |
| 5 | 0.24528 | 0.5295 | -0.792516 | 1.28307 | 0.6432 |
| 6 | 0.20831 | 1.08024 | -1.90892 | 2.32554 | 0.84709 |
| 7 | 0.25615 | 0.87693 | -1.462604 | 1.9749 | 0.77021 |
| ATE | 0.1967 | 0.4483 | -0.6819 | 1.075 | 0.6608 |
| **Intervention Country:** | **Ireland** |  |  |  |  |
| **Leaving out:** | **Italy** |  |  |  |  |
| Post-Intervention period | TE | S.E. | CI.lower | CI.upper | p.value |
| 1 | 0.01539 | 0.12651 | -0.23257 | 0.26334 | 0.9032 |
| 2 | 0.04901 | 0.1685 | -0.28124 | 0.37926 | 0.7712 |
| 3 | 0.07805 | 0.24938 | -0.41073 | 0.56684 | 0.7543 |
| 4 | 0.11332 | 0.32911 | -0.53173 | 0.75837 | 0.7306 |
| 5 | 0.09892 | 0.43084 | -0.7455 | 0.94334 | 0.8184 |
| 6 | 0.31786 | 0.73804 | -1.12868 | 1.7644 | 0.6667 |
| 7 | 0.39397 | 0.63302 | -0.84672 | 1.63467 | 0.5337 |
| ATE | 0.1524 | 0.341 | -0.5159 | 0.8207 | 0.655 |
| Intervention Country: | Ireland |  |  |  |  |
| Leaving out: | Switzerland |  |  |  |  |
| Post-Intervention period | TE | S.E. | CI.lower | CI.upper | p.value |
| 1 | 0.02754 | 0.12183 | -0.21125 | 0.26632 | 0.8212 |
| 2 | 0.06975 | 0.18287 | -0.28868 | 0.42818 | 0.7029 |
| 3 | 0.10719 | 0.26939 | -0.42081 | 0.63519 | 0.6907 |
| 4 | 0.14622 | 0.35757 | -0.5546 | 0.84704 | 0.6826 |
| 5 | 0.13433 | 0.48124 | -0.80889 | 1.07755 | 0.7801 |
| 6 | 0.343 | 0.87062 | -1.36339 | 2.04939 | 0.6936 |
| 7 | 0.42221 | 0.78462 | -1.11562 | 1.96005 | 0.5905 |
| ATE | 0.1786 | 0.4008 | -0.6069 | 0.9641 | 0.6559 |
| **Intervention Country:** | **Ireland** |  |  |  |  |
| **Leaving out:** | **Greece** |  |  |  |  |
| Post-Intervention period | TE | S.E. | CI.lower | CI.upper | p.value |
| 1 | -0.117947 | 0.06969 | -0.254541 | 0.01865 | 0.0905683 |
| 2 | -0.152898 | 0.08644 | -0.322321 | 0.01653 | 0.0769291 |
| 3 | -0.217109 | 0.13183 | -0.475497 | 0.04128 | 0.0995885 |
| 4 | -0.262965 | 0.18065 | -0.617026 | 0.0911 | 0.14548 |
| 5 | -0.384269 | 0.22925 | -0.833599 | 0.06506 | 0.0937046 |
| 6 | -0.55338 | 0.44878 | -1.432979 | 0.32622 | 0.2175509 |
| 7 | -0.517191 | 0.45931 | -1.417418 | 0.38304 | 0.2601558 |
| ATE | -0.3151 | 0.1871 | -0.6818 | 0.05154 | 0.0921 |
| **Intervention Country:** | **Ireland** |  |  |  |  |
| **Leaving out:** | **Sweden** |  |  |  |  |
| Post-Intervention period | TE | S.E. | CI.lower | CI.upper | p.value |
| 1 | -0.007211 | 0.11621 | -0.23498 | 0.22056 | 0.9505 |
| 2 | 0.028121 | 0.18472 | -0.33393 | 0.39018 | 0.879 |
| 3 | 0.048752 | 0.27029 | -0.48102 | 0.57852 | 0.8569 |
| 4 | 0.07413 | 0.35894 | -0.62937 | 0.77763 | 0.8364 |
| 5 | 0.047456 | 0.46059 | -0.85528 | 0.95019 | 0.9179 |
| 6 | 0.245142 | 0.79969 | -1.32222 | 1.81251 | 0.7592 |
| 7 | 0.320143 | 0.71159 | -1.07455 | 1.71483 | 0.6528 |
| ATE | 0.1081 | 0.3822 | -0.6411 | 0.8572 | 0.7774 |
| **Intervention Country:** | **Portugal** |  |  |  |  |
| **Leaving out:** | **Austria** |  |  |  |  |
| Post-Intervention period | TE | S.E. | CI.lower | CI.upper | p.value |
| 1 | -0.09585 | 0.10415 | -0.29997 | 0.10828 | 0.35743 |
| 2 | -0.25872 | 0.14588 | -0.54464 | 0.0272 | 0.07614 |
| 3 | -0.26386 | 0.22091 | -0.69683 | 0.16912 | 0.23232 |
| 4 | -0.26956 | 0.27021 | -0.79917 | 0.26005 | 0.31848 |
| 5 | -0.34084 | 0.29949 | -0.92783 | 0.24615 | 0.25509 |
| 6 | -0.25167 | 0.40188 | -1.03934 | 0.536 | 0.53116 |
| 7 | -0.295 | 0.4542 | -1.18521 | 0.59522 | 0.51602 |
| ATE | -0.2536 | 0.2331 | -0.7105 | 0.2033 | 0.2766 |
| **Intervention Country:** | **Portugal** |  |  |  |  |
| **Leaving out:** | **Germany** |  |  |  |  |
| Post-Intervention period | TE | S.E. | CI.lower | CI.upper | p.value |
| 1 | -0.11039 | 0.09942 | -0.30525 | 0.084473 | 0.26686 |
| 2 | -0.27225 | 0.16058 | -0.58699 | 0.042482 | 0.09 |
| 3 | -0.28758 | 0.23641 | -0.75093 | 0.175767 | 0.22381 |
| 4 | -0.29224 | 0.32183 | -0.92301 | 0.338535 | 0.36385 |
| 5 | -0.35141 | 0.43886 | -1.21157 | 0.508738 | 0.42328 |
| 6 | -0.20304 | 0.79869 | -1.76845 | 1.362374 | 0.79933 |
| 7 | -0.25949 | 0.71328 | -1.65749 | 1.138518 | 0.71601 |
| ATE | -0.2538 | 0.3629 | -0.9651 | 0.4576 | 0.4844 |
| **Intervention Country:** | **Portugal** |  |  |  |  |
| **Leaving out:** | **Netherlands** |  |  |  |  |
| Post-Intervention period | TE | S.E. | CI.lower | CI.upper | p.value |
| 1 | -0.11011 | 0.10585 | -0.31757 | 0.09735 | 0.29821 |
| 2 | -0.27739 | 0.15853 | -0.5881 | 0.03332 | 0.08016 |
| 3 | -0.29539 | 0.22184 | -0.73018 | 0.1394 | 0.183 |
| 4 | -0.30021 | 0.30213 | -0.89238 | 0.29196 | 0.3204 |
| 5 | -0.35653 | 0.428 | -1.19539 | 0.48233 | 0.40484 |
| 6 | -0.21136 | 0.78749 | -1.75481 | 1.33208 | 0.78839 |
| 7 | -0.26272 | 0.72173 | -1.67729 | 1.15186 | 0.71585 |
| ATE | -0.2591 | 0.3612 | -0.9671 | 0.4489 | 0.4732 |
| **Intervention Country:** | **Portugal** |  |  |  |  |
| **Leaving out:** | **Denmark** |  |  |  |  |
| Post-Intervention period | TE | S.E. | CI.lower | CI.upper | p.value |
| 1 | -0.042022 | 0.05237 | -0.14467 | 0.06063 | 0.4224 |
| 2 | -0.197014 | 0.1532 | -0.49727 | 0.10325 | 0.1984 |
| 3 | -0.167578 | 0.21949 | -0.59776 | 0.26261 | 0.4452 |
| 4 | -0.143822 | 0.34555 | -0.82109 | 0.53344 | 0.6773 |
| 5 | -0.196998 | 0.53134 | -1.2384 | 0.8444 | 0.7108 |
| 6 | -0.238619 | 1.06887 | -2.33357 | 1.85633 | 0.8233 |
| 7 | -0.326287 | 0.8752 | -2.04166 | 1.38908 | 0.7093 |
| ATE | -0.1875 | 0.4485 | -1.067 | 0.6917 | 0.676 |
| **Intervention Country:** | **Portugal** |  |  |  |  |
| **Leaving out:** | **Italy** |  |  |  |  |
| Post-Intervention period | TE | S.E. | CI.lower | CI.upper | p.value |
| 1 | -0.12029 | 0.10889 | -0.33371 | 0.093137 | 0.26931 |
| 2 | -0.29227 | 0.15109 | -0.58839 | 0.003856 | 0.05306 |
| 3 | -0.31738 | 0.22229 | -0.75307 | 0.118295 | 0.15335 |
| 4 | -0.32869 | 0.30239 | -0.92136 | 0.263975 | 0.27704 |
| 5 | -0.39532 | 0.4041 | -1.18734 | 0.39671 | 0.32795 |
| 6 | -0.25057 | 0.69101 | -1.60493 | 1.10379 | 0.7169 |
| 7 | -0.31568 | 0.57911 | -1.45071 | 0.819355 | 0.58568 |
| ATE | -0.2886 | 0.3171 | -0.9101 | 0.3329 | 0.3627 |
| **Intervention Country:** | **Portugal** |  |  |  |  |
| **Leaving out:** | **Switzerland** |  |  |  |  |
| Post-Intervention period | TE | S.E. | CI.lower | CI.upper | p.value |
| 1 | -0.10965 | 0.10828 | -0.32187 | 0.10256 | 0.31119 |
| 2 | -0.27073 | 0.15722 | -0.57887 | 0.03741 | 0.08507 |
| 3 | -0.28414 | 0.23373 | -0.74224 | 0.17395 | 0.2241 |
| 4 | -0.28703 | 0.30841 | -0.8915 | 0.31744 | 0.35202 |
| 5 | -0.34711 | 0.40709 | -1.14499 | 0.45078 | 0.39386 |
| 6 | -0.19933 | 0.74379 | -1.65714 | 1.25848 | 0.78871 |
| 7 | -0.25948 | 0.6627 | -1.55834 | 1.03937 | 0.69538 |
| ATE | -0.2511 | 0.3389 | -0.9152 | 0.4131 | 0.4587 |
| **Intervention Country:** | **Portugal** |  |  |  |  |
| **Leaving out:** | **Greece** |  |  |  |  |
| Post-Intervention period | TE | S.E. | CI.lower | CI.upper | p.value |
| 1 | **-0.18845** | 0.06566 | -0.31714 | -0.059763 | 0.004 |
| 2 | **-0.39543** | 0.08138 | -0.55493 | -0.235932 | 0.000 |
| 3 | **-0.46763** | 0.1277 | -0.71792 | -0.217348 | 0.000 |
| 4 | **-0.52182** | 0.17533 | -0.86546 | -0.178169 | 0.003 |
| 5 | **-0.64802** | 0.21492 | -1.06925 | -0.226796 | 0.003 |
| 6 | -0.73836 | 0.40747 | -1.53699 | 0.060261 | 0.070 |
| 7 | -0.82448 | 0.42392 | -1.65535 | 0.006394 | 0.052 |
| ATE | **-0.5406** | 0.1703 | -0.8743 | -0.2069 | 0.001499 |
| **Intervention Country:** | **Portugal** |  |  |  |  |
| **Leaving out:** | **Sweden** |  |  |  |  |
| Post-Intervention period | TE | S.E. | CI.lower | CI.upper | p.value |
| 1 | -0.221319 | 0.11905 | -0.45466 | 0.01202 | 0.06303 |
| 2 | **-0.437659** | 0.1713 | -0.77341 | -0.10191 | 0.01062 |
| 3 | **-0.524999** | 0.25422 | -1.02326 | -0.02674 | 0.03891 |
| 4 | -0.592888 | 0.30947 | -1.19945 | 0.01367 | 0.05539 |
| 5 | **-0.731037** | 0.35457 | -1.42599 | -0.03609 | 0.03923 |
| 6 | -0.831125 | 0.52906 | -1.86807 | 0.20582 | 0.1162 |
| 7 | -0.922114 | 0.55659 | -2.01301 | 0.16878 | 0.09758 |
| ATE | **-0.6087** | 0.2947 | -1.186 | -0.03114 | 0.03886 |
| **Intervention Country:** | **UK** |  |  |  |  |
| **Leaving out:** | **Austria** |  |  |  |  |
| Post-Intervention period | TE | S.E. | CI.lower | CI.upper | p.value |
| 1 | **-0.176412** | 0.07333 | -0.32014 | -0.03268 | 1.61E-02 |
| 2 | **-0.3293817** | 0.11998 | -0.56454 | -0.09422 | 6.05E-03 |
| 3 | **-0.8270356** | 0.16083 | -1.14225 | -0.51182 | 2.71E-07 |
| 4 | **-1.3447215** | 0.22995 | -1.79542 | -0.89402 | 4.98E-09 |
| 5 | **-1.4129164** | 0.27296 | -1.9479 | -0.87793 | 2.26E-07 |
| 6 | **-1.547147** | 0.29451 | -2.12437 | -0.96992 | 1.49E-07 |
| 7 | **-1.6919306** | 0.32815 | -2.33509 | -1.04877 | 2.52E-07 |
| 8 | **-1.7008677** | 0.35282 | -2.39239 | -1.00935 | 1.43E-06 |
| ATE | **-1.129** | 0.2034 | -1.527 | -0.7302 | 2.85E-08 |
| **Intervention Country:** | **UK** |  |  |  |  |
| **Leaving out:** | **Germany** |  |  |  |  |
| Post-Intervention period | TE | S.E. | CI.lower | CI.upper | p.value |
| 1 | **-0.17202** | 0.08644 | -0.34143 | -0.002605 | 4.66E-02 |
| 2 | **-0.324787** | 0.1314 | -0.58232 | -0.067251 | 1.34E-02 |
| 3 | **-0.822386** | 0.18069 | -1.17654 | -0.468235 | 5.33E-06 |
| 4 | **-1.336963** | 0.25819 | -1.843 | -0.830929 | 2.24E-07 |
| 5 | **-1.404724** | 0.32713 | -2.04588 | -0.763566 | 1.75E-05 |
| 6 | **-1.543813** | 0.41331 | -2.35388 | -0.733743 | 1.88E-04 |
| 7 | **-1.704419** | 0.6593 | -2.99662 | -0.412213 | 9.73E-03 |
| 8 | **-1.714628** | 0.58389 | -2.85903 | -0.570224 | 3.32E-03 |
| ATE | **-1.128** | 0.2916 | -1.699 | -0.5565 | 0.0001096 |
| **Intervention Country:** | **UK** |  |  |  |  |
| **Leaving out:** | **Netherlands** |  |  |  |  |
| Post-Intervention period | TE | S.E. | CI.lower | CI.upper | p.value |
| 1 | **-0.179775** | 0.08706 | -0.35041 | -0.009137 | 3.89E-02 |
| 2 | **-0.336254** | 0.12836 | -0.58783 | -0.084678 | 8.80E-03 |
| 3 | **-0.842352** | 0.16257 | -1.16099 | -0.523718 | 2.20E-07 |
| 4 | **-1.36827** | 0.22355 | -1.80642 | -0.930116 | 9.32E-10 |
| 5 | **-1.441897** | 0.2985 | -2.02695 | -0.856848 | 1.36E-06 |
| 6 | **-1.576029** | 0.41002 | -2.37964 | -0.772414 | 1.21E-04 |
| 7 | **-1.722878** | 0.72928 | -3.15224 | -0.293515 | 1.82E-02 |
| 8 | **-1.717003** | 0.61314 | -2.91873 | -0.515279 | 5.11E-03 |
| ATE | **-1.148** | 0.2927 | -1.722 | -0.5744 | 8.75E-05 |
| **Intervention Country:** | **UK** |  |  |  |  |
| **Leaving out:** | **Denmark** |  |  |  |  |
| Post-Intervention period | TE | S.E. | CI.lower | CI.upper | p.value |
| 1 | **-0.1769745** | 0.04452 | -0.26423 | -0.08972 | 7.03E-05 |
| 2 | **-0.3313136** | 0.06291 | -0.45462 | -0.208 | 1.39E-07 |
| 3 | **-0.8294446** | 0.13638 | -1.09674 | -0.56215 | 1.19E-09 |
| 4 | **-1.3482357** | 0.19206 | -1.72467 | -0.9718 | 2.22E-12 |
| 5 | **-1.4180834** | 0.27834 | -1.96363 | -0.87254 | 3.49E-07 |
| 6 | **-1.5539285** | 0.41999 | -2.3771 | -0.73076 | 2.16E-04 |
| 7 | -1.6944265 | 0.89546 | -3.4495 | 0.06065 | 5.85E-02 |
| 8 | **-1.6987853** | 0.71777 | -3.10559 | -0.29198 | 1.79E-02 |
| ATE | **-1.131** | 0.3125 | -1.744 | -0.519 | 0.0002936 |
| **Intervention Country:** | **UK** |  |  |  |  |
| **Leaving out:** | **Italy** |  |  |  |  |
| Post-Intervention period | TE | S.E. | CI.lower | CI.upper | p.value |
| 1 | **-0.172405** | 0.08471 | -0.33843 | -0.006376 | 4.18E-02 |
| 2 | **-0.323885** | 0.13187 | -0.58234 | -0.065433 | 1.40E-02 |
| 3 | **-0.822519** | 0.16983 | -1.15537 | -0.489664 | 1.28E-06 |
| 4 | **-1.337671** | 0.24366 | -1.81524 | -0.860101 | 4.02E-08 |
| 5 | **-1.406093** | 0.31053 | -2.01472 | -0.797465 | 5.95E-06 |
| 6 | **-1.543099** | 0.38756 | -2.30271 | -0.78349 | 6.85E-05 |
| 7 | **-1.706905** | 0.59147 | -2.86616 | -0.547652 | 3.90E-03 |
| 8 | **-1.712665** | 0.4763 | -2.6462 | -0.779129 | 3.24E-04 |
| ATE | **-1.128** | 0.266 | -1.649 | -0.6068 | 2.22E-05 |
| **Intervention Country:** | **UK** |  |  |  |  |
| **Leaving out:** | **Switzerland** |  |  |  |  |
| Post-Intervention period | TE | S.E. | CI.lower | CI.upper | p.value |
| 1 | **-0.174574** | 0.08287 | -0.337 | -0.01215 | 3.52E-02 |
| 2 | **-0.327116** | 0.12921 | -0.58036 | -0.07387 | 1.14E-02 |
| 3 | **-0.824606** | 0.17957 | -1.17656 | -0.47265 | 4.39E-06 |
| 4 | **-1.338318** | 0.26087 | -1.84962 | -0.82702 | 2.89E-07 |
| 5 | **-1.404478** | 0.32632 | -2.04405 | -0.7649 | 1.68E-05 |
| 6 | **-1.540043** | 0.39438 | -2.31302 | -0.76706 | 9.43E-05 |
| 7 | **-1.700224** | 0.59954 | -2.87531 | -0.52514 | 4.57E-03 |
| 8 | **-1.705053** | 0.52524 | -2.7345 | -0.67561 | 1.17E-03 |
| ATE | **-1.127** | 0.2779 | -1.671 | -0.5822 | 5.01E-05 |
| **Intervention Country:** | **UK** |  |  |  |  |
| **Leaving out:** | **Greece** |  |  |  |  |
| Post-Intervention period | TE | S.E. | CI.lower | CI.upper | p.value |
| 1 | **-0.13927** | 0.06849 | -0.27351 | -0.005032 | 4.20E-02 |
| 2 | **-0.27091** | 0.09902 | -0.46498 | -0.076833 | 6.22E-03 |
| 3 | **-0.74688** | 0.12182 | -0.98565 | -0.508111 | 8.74E-10 |
| 4 | **-1.23047** | 0.18005 | -1.58337 | -0.877582 | 8.26E-12 |
| 5 | **-1.27274** | 0.23693 | -1.73712 | -0.808364 | 7.80E-08 |
| 6 | **-1.3796** | 0.28115 | -1.93065 | -0.828552 | 9.25E-07 |
| 7 | **-1.45788** | 0.41567 | -2.27258 | -0.643191 | 4.53E-04 |
| 8 | **-1.45125** | 0.42787 | -2.28986 | -0.612635 | 6.94E-04 |
| ATE | **-0.9936** | 0.1922 | -1.37 | -0.6169 | 2.35E-07 |
| **Intervention Country:** | **UK** |  |  |  |  |
| **Leaving out:** | **Sweden** |  |  |  |  |
| Post-Intervention period | TE | S.E. | CI.lower | CI.upper | p.value |
| 1 | **-0.185297** | 0.09063 | -0.36293 | -0.007661 | 4.09E-02 |
| 2 | **-0.343937** | 0.14062 | -0.61955 | -0.068325 | 1.45E-02 |
| 3 | **-0.837497** | 0.18701 | -1.20404 | -0.470956 | 7.53E-06 |
| 4 | **-1.360575** | 0.26641 | -1.88272 | -0.838426 | 3.27E-07 |
| 5 | **-1.438453** | 0.33209 | -2.08934 | -0.787569 | 1.48E-05 |
| 6 | **-1.584448** | 0.38998 | -2.34879 | -0.82011 | 4.85E-05 |
| 7 | **-1.7225** | 0.5227 | -2.74698 | -0.698021 | 9.83E-04 |
| 8 | **-1.729453** | 0.46845 | -2.64759 | -0.811315 | 2.23E-04 |
| ATE | **-1.15** | 0.2702 | -1.68 | -0.6207 | 2.07E-05 |
| *Bold: denotes statistical significance at an alpha level of 5%. Abbreviations: CS: Synthetic control; ATE: Average treatment effect (across all post-intervention periods); TE: treatment effect (for each time period post-intervention); S.E.: Standard error; CI: Confidence interval.* | | | | | |

| ***eTable 10: Sensitivity analysis of SC and cITS, applying both methods to randomly selected control countries*** | | | | | |
| --- | --- | --- | --- | --- | --- |
| **Germany** | | | | | |
| Post-intervention period | TE | S.E. | CI.lower | CI.upper | p.value |
| 1 | -0.020144 | 0.08268 | -0.18219 | 0.1419 | 0.8075 |
| 2 | -0.013699 | 0.12726 | -0.26313 | 0.23573 | 0.9143 |
| 3 | -0.006988 | 0.19129 | -0.3819 | 0.36793 | 0.9709 |
| 4 | 0.003356 | 0.26307 | -0.51226 | 0.51897 | 0.9898 |
| 5 | -0.055265 | 0.35913 | -0.75914 | 0.64861 | 0.8777 |
| 6 | -0.018484 | 0.6486 | -1.28973 | 1.25276 | 0.9773 |
| 7 | -0.140913 | 0.56864 | -1.25543 | 0.9736 | 0.8043 |
| ATE | -0.03602 | 0.2878 | -0.6002 | 0.5281 | 0.9004 |
| country*post (level change) | 0.003354 | 0.06117594 | -0.1242569 | 0.13096467 | 0.9568 |
| country*year_post (trend change) | **0.074066** | 0.01506048 | 0.04265005 | 0.10548125 | 0.0001 |
| **Denmark** | | | | | |
| Post-intervention period | TE | S.E. | CI.lower | CI.upper | p.value |
| 1 | 0.068993 | 0.11753 | -0.16136 | 0.29935 | 0.5571826 |
| 2 | 0.044709 | 0.16725 | -0.28309 | 0.37251 | 0.7892203 |
| 3 | 0.088902 | 0.24948 | -0.40007 | 0.57788 | 0.7215805 |
| 4 | 0.117013 | 0.31365 | -0.49772 | 0.73175 | 0.7090946 |
| 5 | 0.096472 | 0.38315 | -0.65448 | 0.84742 | 0.8012045 |
| 6 | -0.490478 | 0.48628 | -1.44356 | 0.46261 | 0.3131474 |
| 7 | -0.605453 | 0.47984 | -1.54592 | 0.33501 | 0.2070252 |
| ATE | -0.09712 | 0.3059 | -0.6967 | 0.5024 | 0.7509 |
| country*post (level change) | 0.365944 | 0.18610039 | -0.0222544 | 0.75414284 | 0.0633 |
| country*year_post (trend change) | -0.082338 | 0.04581475 | -0.1779054 | 0.01323036 | 0.0874 |
| **Italy** | | | | | |
| Post-intervention period | TE | S.E. | CI.lower | CI.upper | p.value |
| 1 | -4.04E-02 | 0.13296 | -0.30095 | 0.22025 | 0.7615 |
| 2 | -1.15E-01 | 0.16726 | -0.44289 | 0.21274 | 0.4914 |
| 3 | -1.75E-01 | 0.25593 | -0.6765 | 0.32674 | 0.4944 |
| 4 | -2.16E-01 | 0.31878 | -0.84059 | 0.40902 | 0.4985 |
| 5 | -2.50E-01 | 0.36703 | -0.96888 | 0.46983 | 0.4966 |
| 6 | -2.95E-01 | 0.48308 | -1.24222 | 0.65141 | 0.5409 |
| 7 | -3.35E-01 | 0.50688 | -1.32876 | 0.65816 | 0.5083 |
| ATE | -2.04E-01 | 0.288 | -0.7683 | 0.3608 | 0.4793 |
| country*post (level change) | -0.027364 | 0.03716069 | -0.10488 | 0.05015165 | 0.4701 |
| country*year_post (trend change) | -0.005645 | 0.00914833 | -0.0247277 | 0.01343851 | 0.5442 |
| *Bold: denotes statistical significance at an alpha level of 5%. Abbreviations: CS: Synthetic control; cITS: Controlled interrupted times series; ATE: Average treatment effect (across all post-intervention periods); TE: treatment effect (for each time period post-intervention); S.E.: Standard error; CI: Confidence interval.* | | | | | |

| ***eTable 11: Results of synthetic control analysis: estimated treatment effects of tiered soft drink tax on mean sales-weighted sugar content, using the “augsynth” package in R*** | | |
| --- | --- | --- |
| **France** |  |  |
| *Year after intervention* | *Treatment Effect** | *p-value* |
| 1 year | 0.129 | 0.458 |
| 6 years | -0.128 | 0.459 |
| Average Treatment Effect | 0.0868 | 0.21 |
| **Ireland** |  |  |
| *Year after intervention* | *Treatment Effect** | *p-value* |
| 1 year | 0.101 | 0.144 |
| 7 years | 0.311 | 0.131 |
| Average Treatment Effect | 0.23 | 0.97 |
| **Portugal** |  |  |
| *Year after intervention* | *Treatment Effect** | *p-value* |
| 1 year | -0.079 | 0.122 |
| 7 years | -0.589 | 0.129 |
| Average Treatment Effect | -0.357 | 0.95 |
| **UK** |  |  |
| *Year after intervention* | *Treatment Effect** | *p-value* |
| 1 year | -0.186 | 0.165 |
| 8 years | -1.63 | 0.158 |
| Average Treatment Effect | **-1.12** | **0.014** |
| *Bold: denotes statistical significance at an alpha level of 5 %. Control Countries: Austria, Germany, Netherlands, Denmark, Greece, Italy, Sweden, Switzerland. *in g of sugar/100 ml* | | |

| ***eTable 12: Results of synthetic control analysis: estimated treatment effects of tiered soft drink tax on mean sales-weighted sugar content, assuming a 80% sugar content of syrups*** | | |
| --- | --- | --- |
| **France** |  |  |
|  | *Treatment Effect** | *p-value* |
| Average Treatment Effect | -0.33 | 0.27 |
| **Ireland** |  |  |
|  | *Treatment Effect** | *p-value* |
| Average Treatment Effect | 0.18 | 0.61 |
| **Portugal** |  |  |
|  | *Treatment Effect** | *p-value* |
| Average Treatment Effect | -0.25 | 0.42 |
| **UK** |  |  |
|  | *Treatment Effect** | *p-value* |
| Average Treatment Effect | **-1.10** | **<0,001** |
| *Bold: denotes statistical significance at an alpha level of 5 %. Control Countries: Austria, Germany, Netherlands, Denmark, Greece, Italy, Sweden, Switzerland. *in g of sugar/100 ml* | | |

# Differences between protocol and manuscript

This study is based on a protocol registered with the Open Science Framework (registration <https://doi.org/10.17605/OSF.IO/VEJZU>) before we began the analysis of the data [44]. In the following, we will describe differences between the protocol and this manuscript.

- Upon further research on soft drink taxes in Europe, we discovered that Norway has recently abolished its soft drink tax, contrary to the information documented in the protocol. Nonetheless, it is important to note that this tax was in effect until 2021, rendering Norway ineligible as a control country for this study, as it remained in force for the majority of the period researched.
- Subsequent research revealed the implementation of a tiered soft drink tax system in Croatia. Nevertheless, this finding does not impact the analysis since Croatia only introduced the tax in 2020, resulting in an insufficient amount of post-intervention data for meaningful inclusion in the study.
- As outlined in the paper, we added a post-hoc sensitivity analysis to test whether our specific choice of the gsynth model had an influence on the findings.

# Reporting guideline checklist

The STROBE-nut reporting guideline checklist is available online at: <https://www.strobe-nut.ugent.be/content/recommendations>

Page numbers with a preceding e (e1, e2, etc.) refer to supplementary material.

| ***eTable 13: STROBE-nut reporting guideline*** | | | |  |
| --- | --- | --- | --- | --- |
| **Nr** | **Item** | **STROBE recommendations** | **STROBE-nut** | **Page Nr** |
| 1 | **Title and**  **abstract** | (a) Indicate the study’s design with a commonly used term in the title or the abstract.  (b) Provide in the abstract an informative and balanced summary of what was done and what was found. | **nut-1**State the dietary/nutritional assessment method(s) used in the title, abstract, or keywords. | 1-3 |
|  | **Introduction** |  |  |  |
| 2 | Background rationale | Explain the scientific background and rationale for the investigation being reported. |  | 3-5 |
| 3 | Objectives | State specific objectives, including any pre-specified hypotheses. |  | 3-4 |
|  | **Methods** |  |  |  |
| 4 | Study design | Present key elements of study design early in the paper. |  | 5 |
| 5 | Settings | Describe the setting, locations, and relevant dates, including periods of recruitment, exposure, follow-up, and data collection. | **nut-5** Describe any characteristics of the study settings that might affect the dietary intake or nutritional status of the participants, if applicable. | 5-7 |
| 6 | Participants | a) Cohort study—Give the eligibility criteria, and the sources and methods of selection of participants. Describe methods of follow-up.  Case-control study—Give the eligibility criteria, and the sources and methods of case ascertainment and control selection. Give the rationale for the choice of cases and controls.  Cross-sectional study—Give the eligibility criteria, and the sources and methods of selection of participants.  (b) Cohort study—For matched studies, give matching criteria and number of exposed and unexposed.  Case-control study—For matched studies, give matching criteria and the number of controls per case. | **nut-6** Report particular dietary, physiological or nutritional characteristics that were considered when selecting the target population. | 5-7 |
| 7 | Variables | Clearly define all outcomes, exposures, predictors, potential confounders, and effect modifiers. Give diagnostic criteria, if applicable. | **nut-7.1**Clearly define foods, food groups, nutrients, or other food components.  **nut-7.2** When using dietary patterns or indices, describe the methods to obtain them and their nutritional properties. | 8 |
| 8 | Data sources - measurements | For each variable of interest, give sources of data and details of methods of assessment (measurement).Describe comparability of assessment methods if there is more than one group. | **nut-8.1** Describe the dietary assessment method(s), e.g., portion size estimation, number of days and items recorded, how it was developed and administered, and how quality was assured. Report if and how supplement intake was assessed.  **nut-8.2** Describe and justify food composition data used. Explain the procedure to match food composition with consumption data. Describe the use of conversion factors, if applicable.  **nut-8.3** Describe the nutrient requirements, recommendations, or dietary guidelines and the evaluation approach used to compare intake with the dietary reference values, if applicable.  **nut-8.4** When using nutritional biomarkers, additionally use the STROBE Extension for Molecular Epidemiology (STROBE-ME). Report the type of biomarkers used and their usefulness as dietary exposure markers.  **nut-8.5** Describe the assessment of nondietary data (e.g., nutritional status and influencing factors) and timing of the assessment of these variables in relation to dietary assessment.  **nut-8.6** Report on the validity of the dietary or nutritional assessment methods and any internal or external validation used in the study, if applicable. | 7-8 |
| 9 | Bias | Describe any efforts to address potential sources of bias. | **nut-9** Report how bias in dietary or nutritional assessment was addressed, e.g., misreporting, changes in habits as a result of being measured, or data imputation from other sources | N.A. |
| 10 | Study Size | Explain how the study size was arrived at. |  | 5-6 |
| 11 | Quantitative variables | Explain how quantitative variables were handled in the analyses. If applicable, describe which groupings were chosen and why. | **nut-11** Explain categorization of dietary/nutritional data (e.g., use of N-tiles and handling of nonconsumers) and the choice of reference category, if applicable. | 8 |
| 12 | Statistical Methods | (a) Describe all statistical methods, including those used to control for confounding  (b) Describe any methods used to examine subgroups and interactions.  (c) Explain how missing data were addressed.  (d) Cohort study—If applicable, explain how loss to follow-up was addressed.  Case-control study—If applicable, explain how matching of cases and controls was addressed.  Cross-sectional study—If applicable, describe analytical methods taking account of sampling strategy.  (e) Describe any sensitivity analyses. | **nut-12.1** Describe any statistical method used to combine dietary or nutritional data, if applicable.  **nut-12.2** Describe and justify the method for energy adjustments, intake modeling, and use of weighting factors, if applicable.  **nut-12.3** Report any adjustments for measurement error, i.e,. from a validity or calibration study. | 8-10 |
|  | **Results** |  |  |  |
| 13 | Participants | (a) Report the numbers of individuals at each stage of the study—e.g., numbers potentially eligible, examined for eligibility, confirmed eligible, included in the study, completing follow-up, and analyzed.  (b) Give reasons for non-participation at each stage.  (c) Consider use of a flow diagram. | **nut-13** Report the number of individuals excluded based on missing, incomplete or implausible dietary/nutritional data. | N.A. |
| 14 | Descriptive data | (a) Give characteristics of study participants (e.g., demographic, clinical, social) and information on exposures and potential confounders  (b) Indicate the number of participants with missing data for each variable of interest  (c) Cohort study—Summarize follow-up time (e.g., average and total amount) | **nut-14** Give the distribution of participant characteristics across the exposure variables if applicable. Specify if food consumption of total population or consumers only were used to obtain results. | 10-11 |
| 15 | Outcome data | Cohort study—Report numbers of outcome events or summary measures over time.  Case-control study—Report numbers in each exposure category, or summary measures of exposure.  Cross-sectional study—Report numbers of outcome events or summary measures. |  | 10-11 |
| 16 | Main results | (a) Give unadjusted estimates and, if applicable, confounder-adjusted estimates and their precision (e.g., 95% confidence interval).  Make clear which confounders were adjusted for and why they were included.  (b) Report category boundaries when continuous variables were categorized.  (c) If relevant, consider translating estimates of relative risk into absolute risk for a meaningful time period. | **nut-16** Specify if nutrient intakes are reported with or without inclusion of dietary supplement intake, if applicable. | 11-13 |
| 17 | Other analyses | Report other analyses done—e.g., analyses of subgroups and interactions and sensitivity analyses. | **nut-17**Report any sensitivity analysis (e.g., exclusion of misreporters or outliers) and data imputation, if applicable. | 13 |
|  | **Discussion** |  |  |  |
| 18 | Key results | Summarize key results with reference to study objectives. |  | 14 |
| 19 | Limitation | Discuss limitations of the study, taking into account sources of potential bias or imprecision. Discuss both direction and magnitude of any potential bias. | **nut-19** Describe the main limitations of the data sources and assessment methods used and implications for the interpretation of the findings. | 16-17 |
| 20 | Interpretation | Give a cautious overall interpretation of results considering objectives, limitations, multiplicity of analyses, results from similar studies, and other relevant evidence. | **nut-20** Report the nutritional relevance of the findings, given the complexity of diet or nutrition as an exposure. | 14-16 |
| 21 | Generalizability | Discuss the generalizability (external validity) of the study results. |  | 17-18 |
|  | **Other information** |  |  |  |
| 22 | Funding | Give the source of funding and the role of the funders for the present study and, if applicable, for the original study on which the present article is based. |  | 19 |
|  | *Ethics* |  | **nut-22.1**Describe the procedure for consent and study approval from ethics committee(s). | N.A. |
|  | *Supplementary material* |  | **nut-22.2** Provide data collection tools and data as online material or explain how they can be accessed. | e9-e21 |

References

1. saeima.lv. Saeima increases excise duty for sweetened non-alcoholic beverages. 7/13/2023. <https://www.saeima.lv/en/news/saeima-news/28716-saeima-increases-excise-duty-for-sweetened-non-alcoholic-beverages>. Accessed 13 Jul 2023.

2. Smilović I. Cigarettes, alcohol and soft drinks prices to rise in Croatia - The Dubrovnik Times. The Dubrovnik Times. 3/19/2020.

3. Wierzejska RE. The Impact of the Sweetened Beverages Tax on Their Reformulation in Poland-The Analysis of the Composition of Commercially Available Beverages before and after the Introduction of the Tax (2020 vs. 2021). Int J Environ Res Public Health 2022. doi:10.3390/ijerph192114464.

4. Republique Francaise. Taxation des boissons. 2022. <https://entreprendre.service-public.fr/vosdroits/F32101>. Accessed 27 Nov 2022.

5. Da República D. Código dos Impostos Especiais de Consumo - CIEC - Artigo 87.º-C | DRE. 5/24/2023. <https://dre.pt/dre/legislacao-consolidada/decreto-lei/2010-34478675-185654447>. Accessed 24 May 2023.

6. Institute for Government. Sugar tax | Institute for Government. 5/31/2023. <https://www.instituteforgovernment.org.uk/article/explainer/sugar-tax>. Accessed 31 May 2023.

7. Revenue Irish Tax and Customs. Sugar Sweetened Drinks Tax (SSDT): Rate of Tax. 1/10/2023. <https://www.revenue.ie/en/companies-and-charities/excise-and-licences/sugar-sweetened-drinks-tax/rate-of-tax.aspx>. Accessed 10 Jan 2023.

8. Global Food Research Program. Maps Archives - Sugary drink taxes around the world. University of North Carolina - Chapel Hill. Updated 2023. 2023. <https://www.globalfoodresearchprogram.org/wp-content/uploads/2023/06/GFRP-UNC_Tax_maps_beverages_2023_06.pdf>. Accessed 17 May 2023.

9. Le Bodo Y, Etilé F, Julia C, Friant-Perrot M, Breton E, Lecocq S, et al. Public health lessons from the French 2012 soda tax and insights on the modifications enacted in 2018. Health Policy. 2022;126:585–91. doi:10.1016/j.healthpol.2022.04.012.

10. Crosbie E, Florence D, Nanthaseang M, Godoy L. Examining the policy process of sugar-sweetened beverage taxation in Ireland. Health Policy. 2022;126:738–43. doi:10.1016/j.healthpol.2022.06.002.

11. Houghton F, Moran Stritch J, Nwanze L. An examination of Ireland’s sugar sweetened beverage tax (sugar tax) in practice. J Public Health (Oxf) 2023. doi:10.1093/pubmed/fdad097.

12. Gonçalves J, Merenda R, Pareira dos Santos J. Not So Sweet: The Impact of the Portuguese Soda Tax on Producers: RWI; 2022.

13. Goiana-da-Silva F, Cruz-E-Silva D, Gregório MJ, Miraldo M, Darzi A, Araújo F. The future of the sweetened beverages tax in Portugal. Lancet Public Health. 2018;3:e562. doi:10.1016/S2468-2667(18)30240-8.

14. HM Treasury, HM Revenue & Customs, and Department of Health and Social Care. Soft Drinks Industry Levy: 12 things you should know. 2016. <https://www.gov.uk/government/news/soft-drinks-industry-levy-12-things-you-should-know>. Accessed 9 Nov 2022.

15. Kurz CF, König AN. The causal impact of sugar taxes on soft drink sales: evidence from France and Hungary. Eur J Health Econ. 2021;22:905–15. doi:10.1007/s10198-021-01297-x.

16. Teng AM, Jones AC, Mizdrak A, Signal L, Genç M, Wilson N. Impact of sugar-sweetened beverage taxes on purchases and dietary intake: Systematic review and meta-analysis. Obesity Reviews. 2019;20:1187–204. doi:10.1111/obr.12868.

17. Cawley J, Thow AM, Wen K, Frisvold D. The Economics of Taxes on Sugar-Sweetened Beverages: A Review of the Effects on Prices, Sales, Cross-Border Shopping, and Consumption. Annu Rev Nutr. 2019;39:317–38. doi:10.1146/annurev-nutr-082018-124603.

18. Fletcher JM, Frisvold DE, Tefft N. The effects of soft drink taxes on child and adolescent consumption and weight outcomes. Journal of Public Economics. 2010;94:967–74. doi:10.1016/j.jpubeco.2010.09.005.

19. Royo-Bordonada MÁ, Fernández-Escobar C, Simón L, Sanz-Barbero B, Padilla J. Impact of an excise tax on the consumption of sugar-sweetened beverages in young people living in poorer neighbourhoods of Catalonia, Spain: a difference in differences study. BMC Public Health. 2019;19:1553. doi:10.1186/s12889-019-7908-5.

20. Bonnet C, Réquillart V. Tax incidence with strategic firms in the soft drink market. Journal of Public Economics. 2013;106:77–88. doi:10.1016/j.jpubeco.2013.06.010.

21. Capacci S, Allais O, Bonnet C, Mazzocchi M. The impact of the French soda tax on prices and purchases. An ex post evaluation. PLoS One. 2019;14:e0223196. doi:10.1371/journal.pone.0223196.

22. Cuadrado C, Dunstan J, Silva-Illanes N, Mirelman AJ, Nakamura R, Suhrcke M. Effects of a sugar-sweetened beverage tax on prices and affordability of soft drinks in Chile: A time series analysis. Soc Sci Med. 2020;245:112708. doi:10.1016/j.socscimed.2019.112708.

23. Bergman UM, Lynggård Hansen N. Are Excise Taxes on Beverages Fully Passed Through to Prices? The Danish Evidence. FA. 2019;75:323. doi:10.1628/fa-2019-0010.

24. Cabrera Escobar MA, Veerman JL, Tollman SM, Bertram MY, Hofman KJ. Evidence that a tax on sugar sweetened beverages reduces the obesity rate: a meta-analysis. BMC Public Health. 2013;13:1072. doi:10.1186/1471-2458-13-1072.

25. Briggs ADM, Mytton OT, Kehlbacher A, Tiffin R, Rayner M, Scarborough P. Overall and income specific effect on prevalence of overweight and obesity of 20% sugar sweetened drink tax in UK: econometric and comparative risk assessment modelling study. BMJ. 2013;347:f6189. doi:10.1136/bmj.f6189.

26. Backholer K, Sarink D, Beauchamp A, Keating C, Loh V, Ball K, et al. The impact of a tax on sugar-sweetened beverages according to socio-economic position: a systematic review of the evidence. Public Health Nutr. 2016;19:3070–84. doi:10.1017/S136898001600104X.

27. Fernandez MA, Raine KD. Insights on the Influence of Sugar Taxes on Obesity Prevention Efforts. Curr Nutr Rep. 2019;8:333–9. doi:10.1007/s13668-019-00282-4.

28. Public Health England. Sugar Reduction: Report on Progress between 2015 and 2019. September 2019. <https://assets.publishing.service.gov.uk/government/uploads/system/uploads/attachment_data/file/839756/Sugar_reduction_yr2_progress_report.pdf>. Accessed 13 Jul 2023.

29. Bandy LK, Scarborough P, Harrington RA, Rayner M, Jebb SA. Reductions in sugar sales from soft drinks in the UK from 2015 to 2018. BMC Med. 2020;18:20. doi:10.1186/s12916-019-1477-4.

30. Hashem KM, He FJ, MacGregor GA. Labelling changes in response to a tax on sugar-sweetened beverages, United Kingdom of Great Britain and Northern Ireland. Bull World Health Organ. 2019;97:818–27. doi:10.2471/BLT.19.234542.

31. Scarborough P, Adhikari V, Harrington RA, Elhussein A, Briggs A, Rayner M, et al. Impact of the announcement and implementation of the UK Soft Drinks Industry Levy on sugar content, price, product size and number of available soft drinks in the UK, 2015-19: A controlled interrupted time series analysis. PLoS Med. 2020;17:e1003025. doi:10.1371/journal.pmed.1003025.

32. Chu BTY, Irigaray CP, Hillier SE, Clegg ME. The sugar content of children’s and lunchbox beverages sold in the UK before and after the soft drink industry levy. Eur J Clin Nutr. 2020;74:598–603. doi:10.1038/s41430-019-0489-7.

33. Goiana-da-Silva F, Nunes AM, Miraldo M, Bento A, Breda J, Araújo FF. Fiscalidade ao Serviço da Saúde Pública: A Experiência na Tributação das Bebidas Açucaradas em Portugal. [Using Pricing Policies to Promote Public Health: The Sugar Sweetened Beverages Taxation Experience in Portugal]. Acta Med Port. 2018;31:191–5. doi:10.20344/amp.10222.

34. Stacey N, Mudara C, Ng SW, van Walbeek C, Hofman K, Edoka I. Sugar-based beverage taxes and beverage prices: Evidence from South Africa’s Health Promotion Levy. Soc Sci Med. 2019;238:112465. doi:10.1016/j.socscimed.2019.112465.

35. Allais O, Enderli G, Sassi F, Soler L-G. Effective policies to promote sugar reduction in soft drinks: lessons from a comparison of six European countries. Eur J Public Health 2023. doi:10.1093/eurpub/ckad157.

36. EU Vocabularies. 7206 Europe - EU Vocabularies - Publications Office of the EU. 5/31/2023. <https://op.europa.eu/en/web/eu-vocabularies/concept-scheme/-/resource?uri=http://eurovoc.europa.eu/100277>. Accessed 31 May 2023.

37. Thow AM, Rippin HL, Mulcahy G, Duffey K, Wickramasinghe K. Sugar-sweetened beverage taxes in Europe: learning for the future. Eur J Public Health. 2022;32:273–80. doi:10.1093/eurpub/ckab211.

38. The Norwegian Tax Administration. Excise duty on non-alcoholic beverages. 7/17/2023. <https://www.skatteetaten.no/en/business-and-organisation/vat-and-duties/excise-duties/about-the-excise-duties/alkoholfrie-drikkevarer/>. Accessed 17 Jul 2023.

39. Lopez Bernal J, Cummins S, Gasparrini A. The use of controls in interrupted time series studies of public health interventions. Int J Epidemiol. 2018;47:2082–93. doi:10.1093/ije/dyy135.

40. Lopez Bernal J, Cummins S, Gasparrini A. Interrupted time series regression for the evaluation of public health interventions: a tutorial. Int J Epidemiol. 2017;46:348–55. doi:10.1093/ije/dyw098.

41. Euromonitor. Passport. 2/10/2023. <https://www.euromonitor.com/our-expertise/passport>. Accessed 10 Feb 2023.

42. BMJ. Verordnung über einige zur menschlichen Ernährung bestimmte Zuckerarten (Zuckerartenverordnung), Anlage 1 (zu den §§ 1 bis 4) - Bezeichnungen und Begriffsbestimmungen. 2003. <https://www.gesetze-im-internet.de/zuckartv_2003/anlage_1.html>. Accessed 18 Jul 2023.

43. Südzucker. Glukose-Fruktose-Sirup. 7/18/2023. <https://www.suedzucker.com/de/product/glukose-fruktose-sirup/>. Accessed 18 Jul 2023.

44. Leibinger A, Huizinga O, Emmert-Fees K, Pedron S, Laxy M, Rehfuess E, et al. The impact of tiered soft drink taxes in Europe on the sugar content of soft drinks: protocol for a synthetic control study: OSF; 2023.
